# Decoding connections in the European population: serum uric acid, sex hormone-binding globulin, total testosterone, estradiol, and female infertility – advanced bidirectional and mediative Mendelian randomization

**DOI:** 10.3389/fendo.2024.1398600

**Published:** 2024-06-28

**Authors:** Zilong Tan, Jianwu Shen, Yuxiao Huang, Junru Li, Mengdi Ding, Aochuan Sun, Jing Hong, Yan Yang, Sheng He, Xueying Zhu, Ran Luo

**Affiliations:** ^1^ Department of Urology, Xiyuan Hospital, China Academy of Chinese Medical Sciences, Beijing, China; ^2^ Department of Urology, Qinghai Hospital of Traditional Chinese Medicine, Xining, China; ^3^ Department of Gynecology, Xiyuan Hospital, China Academy of Chinese Medical Sciences, Beijing, China; ^4^ Department of Internal Medicine, Qinghai Hospital of Traditional Chinese Medicine, Xining, China; ^5^ Department of Geriatrics, Xiyuan Hospital, China Academy of Chinese Medical Sciences, Beijing, China; ^6^ School of Basic Medical Sciences, Peking University, Beijing, China; ^7^ Department of Critical Care Medicine, Affiliated Hospital of Nanjing University of Chinese Medicine, Nanjing, China; ^8^ The First Clinical Medical College, Anhui University of Traditional Chinese Medicine, Hefei, China

**Keywords:** mediated Mendelian randomization, female infertility, endocrinology, metabolism, genome wide association, European

## Abstract

**Background:**

Despite observational links between serum uric acid (SUA), sex hormone-related phenotypes, and female infertility, the causality behind these associations remains uncertain.

**Objective:**

This study utilizes Bidirectional Two-Sample and Mediation Mendelian Randomization to explore the causal relationships and mediation effects of sex hormone-binding globulin (SHBG), total testosterone (TT), and estradiol on these associations.

**Methods:**

We analyzed single-nucleotide polymorphisms (SNPs) associated with SUA and sex hormone levels using data from large-scale GWAS of European populations. Female infertility data were sourced from 6,481 cases and 75,450 controls in the FinnGen Consortium. We employed methods including Inverse Variance Weighted (IVW), Weighted Median, and MR-Egger regression to assess causality.

**Results:**

We found that elevated SUA levels causally increase the risk of female infertility (IVW OR: 1.13, P=0.047). Elevated SUA levels significantly decrease SHBG levels (β=-0.261; P=2.177e-04), with SHBG mediating 27.93% of the effect of SUA on infertility (OR=0.854; 95%CI, 0.793–0.920; P=2.853e-05). Additionally, elevated TT levels, which were associated with decreased SUA levels (β=-0.127), showed an indirect effect on infertility mediated by SUA (β=-0.0187; 95% CI, -0.041 to -0.003; P=0.046).

**Conclusion:**

Our findings demonstrate causal links between high SUA and increased risk of female infertility mediated by hormonal factors such as SHBG and TT. These insights suggest new avenues for infertility treatment and highlight the need for further research into these mechanisms.

## 1 Introduction

The initiation of diagnostic protocols for female infertility is generally recommended after one year of regular, unprotected intercourse without conception, with this period reduced to six months for women over 35. For those over 40, more immediate evaluations are suggested, emphasizing the urgency in these cases (1). Globally, infertility poses a significant public health challenge, affecting an estimated 8–15% of couples of reproductive age. This issue is not just widespread but also escalating, as evidenced by an increase in the age-standardized prevalence of infertility from 1990 to 2017, marked at 0.370% annually in women (2, 3). Beyond the emotional and psychological strain, female infertility contributes to broader societal challenges, impacting psychological well-being, social dynamics, and economic burdens, thereby influencing national fertility rates (4). Furthermore, it is associated with an increased risk of gynecologic cancers and a higher mortality rate, adding to its health implications (5, 6).

The primary physiological causes of female infertility include ovulatory dysfunctions and tubal disorders, notably conditions such as Polycystic Ovary Syndrome (PCOS) and endometriosis (7–9). In recent years, oxidative stress and inflammation have been recognized as key factors in infertility, affecting oocyte aging, follicular development, and fertilization rates (10–12). In this realm, uric acid, a byproduct of purine metabolism, stands out as a significant marker for oxidative stress and inflammation, influencing various organ systems and disease processes (13). While some evidence links uric acid to pro-inflammatory properties and associations with pregnancy complications (14, 15), other studies suggest a protective antioxidant role at certain concentrations (16, 17). The relationship between SUA and infertility is further complicated by the involvement of factors like Sex Hormone-Binding Globulin (SHBG) and reproductive hormones, especially in conditions like PCOS, where evidence remains conflicting (18, 19). This necessitates a deeper investigation into the interactions between these hormonal elements and uric acid, particularly among non-childbearing populations.

Given the ethical and practical challenges associated with conducting Randomized Controlled Trials (RCTs) in the study of genetic influences on diseases, and the inherent limitations of observational studies which often struggle to adequately control for confounding factors, Mendelian Randomization offers a compelling alternative. MR utilizes genetic variants, specifically SNPs, as instrumental variables to create a form of natural randomization that mimics the random assignment found in RCTs. This methodology leverages the principle that alleles are randomly assorted during gamete formation and passed on to offspring independently of confounding environmental and behavioral factors. As such, MR provides a more reliable assessment of causal relationships between exposure factors and outcomes by utilizing these genetic variants that are associated with modifiable exposures (such as serum uric acid or sex hormone levels) to estimate their effect on disease outcomes (such as female infertility) (20, 21). This approach not only helps to overcome the biases often encountered in traditional observational studies but also enhances the validity of the causal inferences drawn from the data.

## 2 Materials and methods

### 2.1 Data sources and availability

In this study, we implemented a mediated MR analysis to discern the genetic influences on both exposure and outcomes, drawing data from multiple datasets (22). This approach was selected to address confounding issues, especially those associated with population stratification. To ensure specificity, our analysis was confined to individuals of European ancestry. Expanding the genetic association mapping to include non-European populations, a significant initiative by Saori Sakaue et al. (23) involved conducting 220 deep phenotype genome-wide association studies (GWAS) within the Japan Biobank, comprising 220,179 participants. This was complemented by meta-analyses incorporating data from the UK Biobank and FinnGen, totaling 628,000 subjects. This extensive collaboration led to the discovery of around 5,000 novel loci. For our study, we utilized a GWAS meta-analysis focusing on serum uric acid levels (ID: ebi-a-GCST90018977), encompassing 343,836 subjects, to obtain a comprehensive genetic association estimate. Similarly, estimates of genetic association with female infertility were extracted from a robust GWAS meta-analysis (ID: finn-b-N14_FEMALEINFERT, Build: HG19/GRCh37), involving 6,481 female infertility patients (Female infertility is defined as the inability of a woman to conceive after one year of regular, unprotected sexual intercourse, or within six months for women over 35 years of age, due to various etiologies, including PCOS, insufficient ovarian reserve, endometriosis, fallopian tube disease, etc) and 68,969 controls across Europe in 2021. At the outset, we identified three mediator variables–Sex Hormone-Binding Globulin, Total Testosterone, and Estradiol–owing to their significant roles in relation to uric acid and female infertility. The summary statistics for these mediators were sourced from GWAS datasets: SHBG levels (ID: ebi-a-GCST90025958), Total Testosterone levels (ID: ebi-a-GCST90012114), and Estradiol levels (ID: ebi-a-GCST90012105), including 397,043 participants with 4,217,370 SNPs, 425,097 participants with 16,132,861 SNPs, and 206,927 participants with 16,136,413 SNPs, respectively. **Table 1** presents the GWAS summaries for each phenotype under investigation. For further details, GWAS summary statistics are accessible at the GWAS Catalog, available online at https://gwas.mrcieu.ac.uk/. Ethical approval: This study only used published or publicly available data (the original GWAS studies). Ethical approval for each study included in the investigation can be found in the original publications (including informed consent from each participant).

**Table 1:** Baseline characteristics of Serum uric acid levels, sex hormone-related phenotypes (Sex hormone-binding globulin, Total testosterone, Estradiol) and female infertility datasets.

Trait	ID	Year	PMID	Population	Sample Size	n SNPs
**Serum uric acid levels**	**ebi-a-GCST90018977**	**2021**	**34594039**	**European**	**343,836**	**19,041,286**
**Sex hormone-binding globulin levels**	**ebi-a-GCST90025958**	**2021**	**34226706**	**European**	**397,043**	**4,217,370**
**Total testosterone levels**	**ebi-a-GCST90012114**	**2020**	**32042192**	**European**	**425,097**	**16,132,861**
**Estradiol levels**	**ebi-a-GCST90012105**	**2020**	**32042192**	**European**	**206,927**	**16,136,413**
**Female infertility**	**finn-b-N14_FEMALEINFERT**	**2021**	**NA**	**European**	**75450**	**16,377,038**

NA, Not Applicable.

### 2.2 Selection of genetic instruments and data harmonization

**Figure 1:**
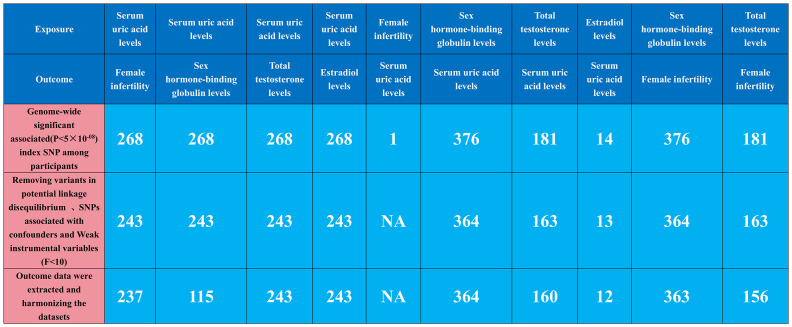
Genetic instrument selection of single-variable Mendelian randomization study.

Our MR analysis began by identifying independent SNPs linked to our factors of interest. We validated three hypotheses to ensure these SNPs were relevant and independent as instrumental variables, selecting them based on genome-wide significance (P<5e-08) and ensuring they were free from confounding factors. To maintain their near-independence, we applied a strict linkage disequilibrium (LD) criterion (r^2^ < 0.001) across 10,000 kilobase pairs, using the LD reference panel from the European population in the 1000 Genomes Project and focusing on bi-allelic SNPs with minor allele frequencies over 0.01. We extracted key summary data from the GWAS for each exposure and outcome, including SNP counts, alleles, effect allele frequency, sample size, case/control numbers, beta coefficients, standard errors, and p-values. When certain SNPs were missing in the outcome GWAS, we used surrogate SNPs in linkage with the original SNPs. This harmonization ensured our genetic variant association estimates matched the effects of the same alleles across datasets. We rigorously selected instrumental variables with an F-statistic over 10 to minimize bias, and used allele frequency data to determine the orientation of alleles in both exposure and outcome GWAS. Detailed information on the SNPs used as instruments in our MR analyses, including proxies for unavailable SNPs in the outcome dataset, is provided in **Figure 1**, **Supplementary Tables S1**–**S10**.

### 2.3 Statistical analyses

#### 2.3.1 Bidirectional and mediated Mendelian randomization

**Figure 2:**
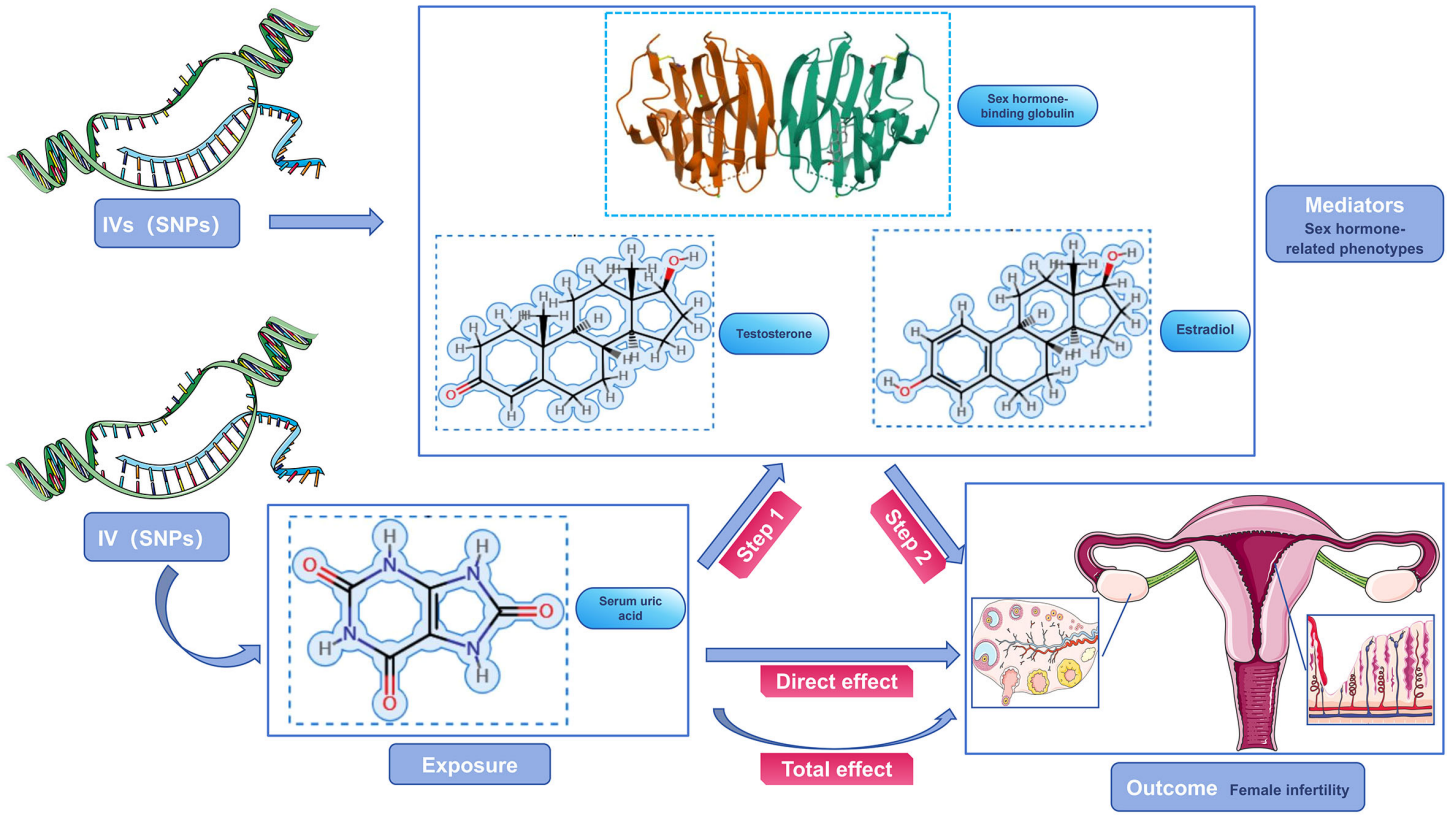
Schematic diagram of the study design. SUA was used as an exposure factor, sex hormone-related phenotypes was used as the mediators, and female infertility was used as an outcome factor; IV, instrument variable.

**Figure 3:**
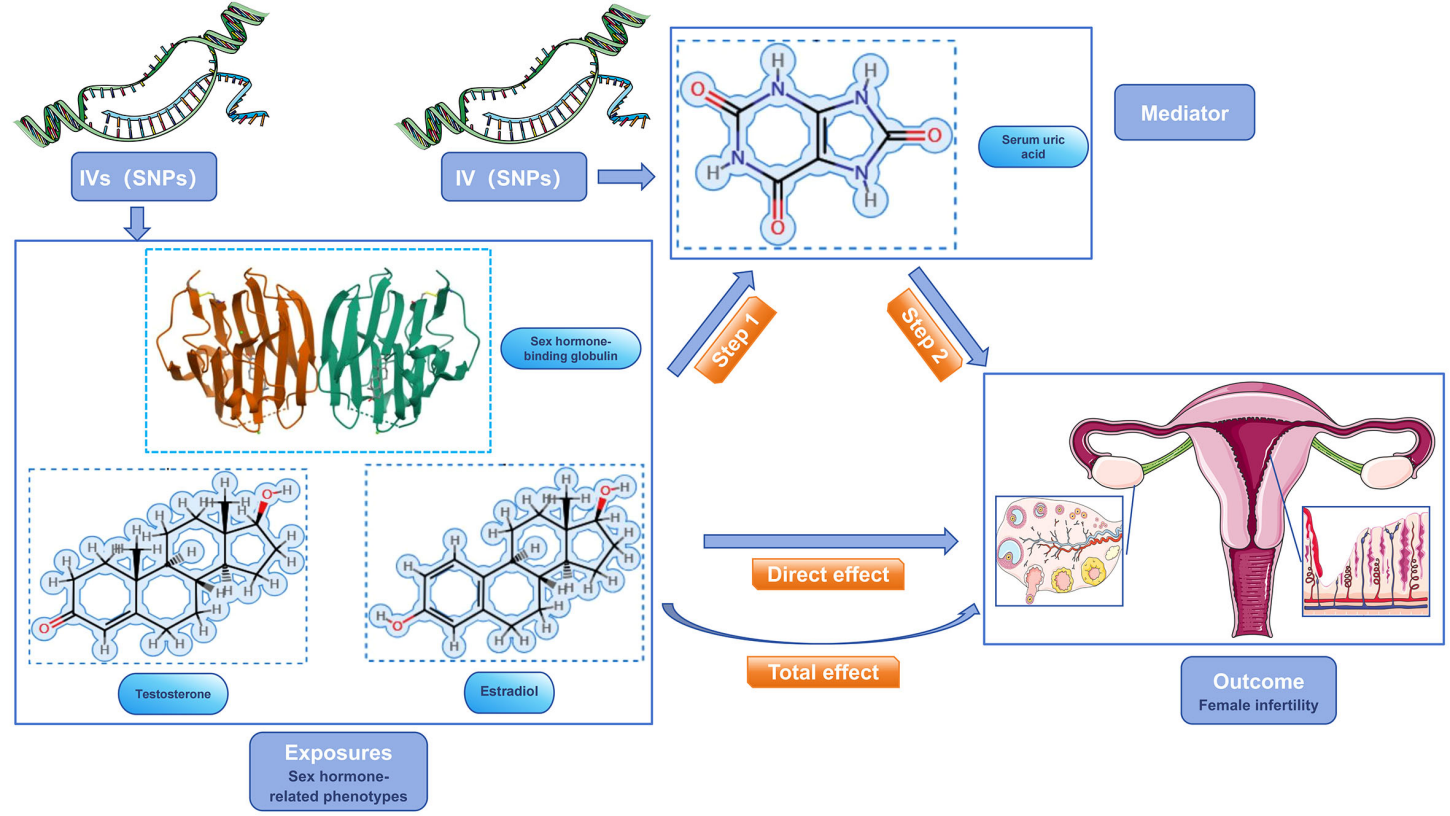
Schematic diagram of the study design. sex hormone-related phenotypes were used as the exposure factors, SUA was used as the mediator, and female infertility was used as an outcome factor; IV, instrument variable.

Our study focused on mediator MR analysis to unravel the causal relationships between SUA, sex hormone-related phenotypes, and female infertility, particularly emphasizing the roles within the mediating pathways. Initially, we conducted a bidirectional two-sample MR analysis, with SUA as the exposure and female infertility as the outcome, to verify any causal link between them. Upon finding a unidirectional causal relationship between SUA and female infertility, we further explored this link using a unidirectional two-sample MR analysis. Here, SUA was the exposure, and the three sex hormone-related phenotypes—SHBG, TT, and Estradiol—acted as mediators. Each of these phenotypes was examined both as a mediator and as an outcome in relation to SUA and infertility. Additionally, we conducted a reverse MR analysis to assess potential reverse causality, examining if sex hormone-related phenotypes could inversely affect SUA levels. This dual approach, comprising both forward and reverse analyses, aimed to not only confirm the mediating roles of these phenotypes but also to explore the possibility of reverse causation. Our approach aimed to understand the complex interplay between SUA and sex hormone-related phenotypes in female infertility, considering both direct and indirect effects (**Figures 2**, **3**). Identifying any reverse causality would further clarify SUA’s role in the context of female reproductive health.

#### 2.3.2 Sensitivity analyses

Our MR analysis utilized TwoSampleMR version 0.5.8 within the R 4.2.3 framework (https://github.com/MRCIEU/TwoSampleMR). We employed five MR methods for a thorough causality assessment, with IVW as the primary method. IVW assigns weights based on standard errors to manage data heterogeneity (24). To ensure robustness and address potential MR assumptions, this was complemented by four additional methods: MR-Egger, Weighted Median, Simple Mode, and Weighted Mode, each contributing to the validation and reduction of data heterogeneity or instrumental variable weaknesses. Sensitivity analyses included Cochran’s Q test for detecting heterogeneity among causal estimates and MR-Egger regression for adjusting potential pleiotropic effects, especially directional pleiotropy influencing the outcome (25). We also generated funnel plots to assess the precision of causal estimates, expecting symmetry around the IVW estimates. Additionally, leave-one-out sensitivity analyses were conducted to determine the influence of individual SNPs on causal effect estimates (26). These analyses collectively reinforced the reliability and robustness of our MR findings.

## 3 Results

### 3.1 Relationship between SUA levels mediated by SHBG and female infertility

#### 3.1.1 Genetically predicted SUA levels are positively associated with the risk of developing female infertility

**Figure 4:**
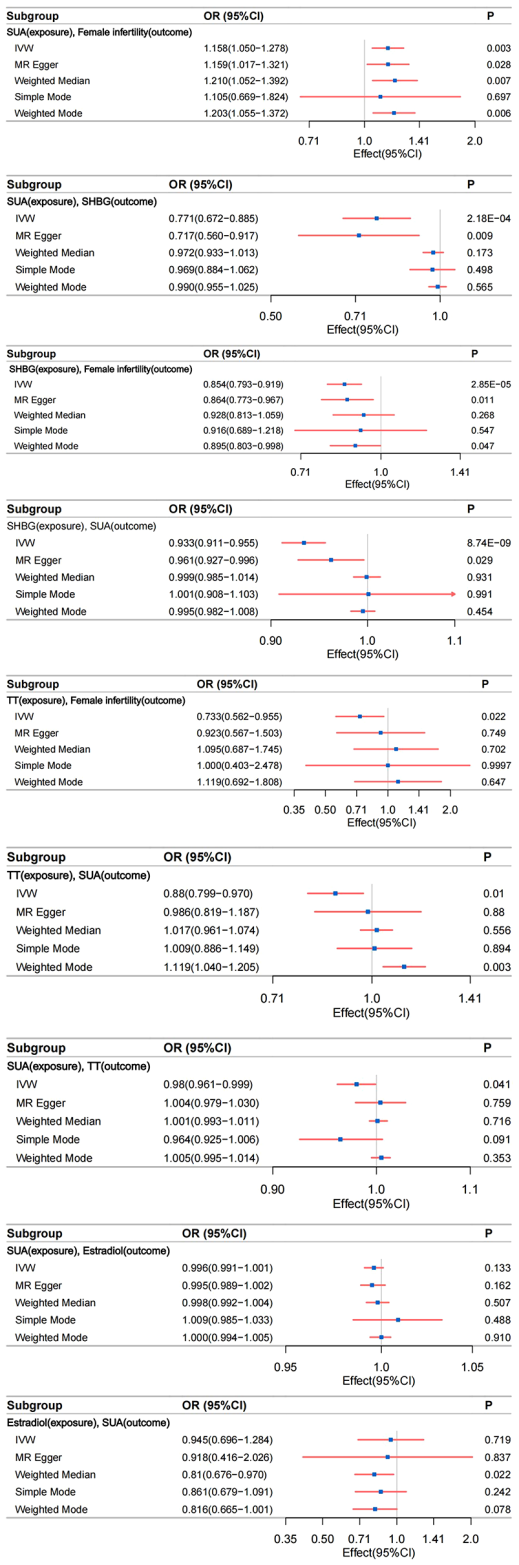
The relationship between different phenotypes was analyzed by bidirectional two-sample and mediated Mendelian randomization; IVW, inverse-variance weighting.

Robust evidence from various MR methods consistently supported a causal relationship between genetically proxied SUA levels and female infertility (**Figure 4**). The primary IVW analysis revealed a positive correlation between SUA levels and female infertility (**Supplementary Figure S1A**), demonstrating a substantial difference (odds ratio [OR]: 1.158; 95% confidence interval [CI]: 1.050–1.278; P=0.003). Specifically, for each standard deviation increase in genetically predicted SUA levels, the primary IVW method indicated a 15.81% escalation in the risk of developing infertility in women. **Supplementary Figure S2A**, **Supplementary Table S11** outline results in alignment with IVW analyses, notably showcasing considerable statistical disparities, particularly evident in MR Egger, Weighted Median, and Weighted Mode analyses. Noteworthy, the Cochran’s Q statistic in the IVW analysis failed to evidence greater heterogeneity in variant-specific causality estimates than expected by chance (Q = 229.664, P=0.438). Additionally, MR-Egger regression analysis exhibited no indication of directional multidirectional effects among genetic variants (Egger intercept=-2.374e-05, P=0.991) (**Supplementary Tables S20**, **S21**). Furthermore, leave-one-out sensitivity analyses highlighted that no single SNP substantially influenced the association between SUA levels and female infertility, as depicted in **Supplementary Figure S3A**. Our analytical approach’s stability was further supported by funnel plots, as presented in **Supplementary Figure S4A**.

In an effort to explore reverse causality, a reverse MR analysis was performed, using genetic susceptibility to female infertility as the exposure factor and SUA levels as the outcome factor. As anticipated, this analysis revealed no evidence of reverse causality between genetically predicted SUA levels and female infertility. This comprehensive examination fortifies the assertion of a unidirectional causal link from SUA levels to increased female infertility risk.

#### 3.1.2 Genetically predicted SUA levels are negatively correlated with SHBG levels

Compelling evidence supports a causal relationship between genetic proxies for SUA levels and SHBG. IVW analyses revealed a significant negative correlation between SUA levels and SHBG (**Supplementary Figure S1B**), with a notable difference (β=-0.261; 95%CI: -0.399 to -0.123; P=2.177e-04). This observation was consistently corroborated by the multidirectional robust approach, as evidenced in **Supplementary Figure S2B**, **Supplementary Table S12**. Notably, MR Egger analysis demonstrated a large statistical difference (β=-0.333; 95%CI: -0.580 to -0.086; P=0.009). Cochran’s Q statistic in analyses did not provide evidence that the heterogeneity of variant-specific causality estimates was less than the heterogeneity of the expected values by chance (Q = 5648.932, P<0.05). Analyses using MR-Egger regression similarly showed no evidence of directional multidirectional effects between genetic variants (Egger intercept = 0.002, P=0.488) (**Supplementary Tables S20**, **S21**). Leave-one-out sensitivity analysis and funnel plots validated the stability of this relationship (**Supplementary Figures S3B**, **S4B**).

#### 3.1.3 Genetically predicted SHBG levels are negatively associated with female infertility

IVW analysis indicated a significant association between genetically predicted SHBG levels and female infertility. The OR was 0.854 (95% CI: 0.793–0.919; P=2.853e-05), suggesting a 14.60% increased risk of infertility per standard deviation increase in SHBG levels (**Supplementary Figure S1C**). This association was consistent across various MR methods, including MR Egger and Weighted Mode analyses (**Supplementary Table S13**, **Supplementary Figure S2C**). Cochran’s Q statistic (Q=380.858, P=0.194) and MR-Egger regression (Egger intercept=-5.368e-04, P=0.782) revealed no significant heterogeneity or directional pleiotropy. The leave-one-out sensitivity analysis and funnel plots further underscored the robustness of this finding (**Supplementary Figures S3C**, **S4C**).

#### 3.1.4 Inverse MR of genetically predicted SUA levels to SHBG

We identified a statistically significant association between SHBG and increased SUA levels (IVW β=-0.070; 95%CI: -0.093 to -0.046; P=8.740e-09) (**Supplementary Figure S1D**). Consistent results were observed with other analytical methods, as outlined in **Supplementary Table S14**, **Supplementary Figure S2D**. Notably, heterogeneity surfaced in the IVW analysis (Q=4181.308, P<0.05). However, it is crucial to acknowledge that MR-Egger regression analysis indicated directional multiple effects within genetic variants, suggesting potential level pleiotropy (Egger intercept=-0.001, P=0.029) (**Supplementary Tables S20**, **S21**). This finding implies a multidirectionality of causality between SHBG and SUA levels, contradicting our initial hypotheses. Consequently, the conclusion regarding the association between SHBG and increased SUA levels should be interpreted with caution due to a lower degree of confidence. Although our study prompts discussion on this conclusion, it does not imply a reverse causal association between SHBG (Exposure factor) and increased SUA levels (Outcome factor). Furthermore, sensitivity analyses excluding one variant suggested that the association is not predominantly driven by any single SNP, as depicted in **Supplementary Figure S3D**, the funnel plot presented in **Supplementary Figure S4D** emphasizes the relative stability.

### 3.2 Relationship between SUA levels mediated by TT and female infertility

#### 3.2.1 Genetically predicted TT levels are negatively associated with the risk of female infertility

Our MR analyses identified a negative association between genetically predicted TT levels and the risk of female infertility. The IVW method indicated an OR of 0.733 (95% CI: 0.562–0.955; P=0.022), suggesting a 26.70% decrease in infertility risk per standard deviation increase in TT levels (**Supplementary Figure S1E**). This finding was consistent across different MR methods (**Supplementary Table S15**, **Supplementary Figure S2E**). Cochran’s Q statistic (Q=169.638, P=0.130) and MR-Egger regression (Egger intercept= -4.003e-03, P= 0.269) indicated no significant heterogeneity or directional effects (**Supplementary Tables S20**, **S21**). Leave-one-out sensitivity analyses and funnel plots reinforced the robustness of this association (**Supplementary Figures S3E**, **S4E**).

Considering that the identified causal relationship between TT and female infertility contradicts current clinical understanding and may be subject to instability, Steiger revalidation was performed on the screened SNPs from the two-sample MR analysis. Steiger filtering was also applied to identify SNPs associated with female infertility as an outcome, aiming to filter the outcome data for SNPs significantly associated with exposure. Validation revealed that no SNPs needed to be filtered among the SNPs filtered (number of outcome SNPs after filtering: 157). However, three SNPs removed during data harmonization (rs10892924, rs11888201, and rs9399469) underwent re-MR analysis. The IVW analysis showed that TT levels remained negatively correlated with SUA levels, with a stronger correlation than in the pre-Steiger validation data ([OR]: -0.350; SE: 0.135; P=0.009) (**Supplementary Figures S1F**–**S4F**). A reverse MR analysis found no evidence of reverse causality between TT levels and female infertility.

#### 3.2.2 Genetically predicted TT levels are negatively correlated with SUA levels

MR study demonstrated a significant negative correlation between genetically proxied TT levels and SUA levels. IVW analyses revealed a negative correlation between TT levels and SUA levels (**Supplementary Figure S1G**), with a statistically significant difference (β=-0.127; 95%CI: -0.225 to -0.030; P=0.010) **Supplementary Table S16**, **Supplementary Figure S2G**. Cochran’s Q in IVW analyses found no evidence of less heterogeneity in variant-specific causality estimates than expected by chance (Q=2396.029, P<0.05). MR-Egger regression showed no directional multidirectional effects between genetic variants (Egger intercept=-0.002, P=0.163) (**Supplementary Tables S20**, **S21**). Leave-one-out sensitivity analyses and funnel plots affirmed the stability of this relationship (**Supplementary Figures 3G**, **S4G**).

#### 3.2.3 Inverse Mendelian randomization of genetically predicted TT levels to SUA levels

We observed a statistically significant association between elevated SUA levels and increased TT levels (IVW β=-0.020; 95% CI: -0.040 to -0.001; P= 0.041) (**Supplementary Figure S1H**). Similar results were obtained with alternative analysis methods, detailed in **Supplementary Table S17**, **Supplementary Figure S2H**. While evidence of heterogeneity emerged in the IVW analysis (Q= 2052.540, P=4.480e-288), it is crucial to note that MR-Egger regression analysis suggested directional effects within genetic variants, indicating potential pleiotropy (Egger intercept=-0.001, P=0.005) (**Supplementary Tables S20**, **S21**). This implies a multidirectional causality between SUA (Exposure factor) and TT (Outcome factor), contradicting our initial three hypotheses. We assert that our study does not support the influence of SUA levels on TT levels. In leave-one-out sensitivity analyses, the overall IVW estimate remained unchanged after excluding any variants (**Supplementary Figure S3H**), funnel plots showed little evidence of departures from symmetry (**Supplementary Figure S4H**), suggesting the need for cautious interpretation of the association between SUA and TT levels.

### 3.3 Alterations in estradiol levels are not involved in the causal relationship between SUA levels and female infertility

#### 3.3.1 Absence of causal relationship between genetically predicted SUA levels and estradiol levels

Our study did not find a significant association between SUA levels and estradiol levels using the primary IVW method (β=-0.004; 95% CI: -0.009 to 0.001; P=0.133) (**Supplementary Figure S1I**). This lack of association was consistent across various MR methods (**Supplementary Table S18**, **Supplementary Figure S2I**). Despite the observed heterogeneity in the IVW analysis (Q=310.243, P=5.278e-04), MR-Egger regression analysis showed no evidence of pleiotropic effects (Egger intercept=4.351e-05, P=0.675) (**Supplementary Tables S20**, **S21**). Leave-one-out sensitivity analyses and the funnel plot confirmed the stability of these findings, indicating that no single SNP primarily drove the non-association (**Supplementary Figures S3I**, **S4I**). Continuing with a reverse Mendelian analysis, using genetic susceptibility to estradiol levels as the exposure and SUA levels as the outcome, we found no significant association (IVW β=-0.056; 95% CI: -0.362 to 0.250; P=0.719) (**Supplementary Figure S1J**). This result was corroborated by other MR methods (**Supplementary Table S19**, **Supplementary Figure S2J**). The Cochran’s Q statistic indicated evidence of heterogeneity in the IVW analysis (Q=54.911, P=8.047e-08). MR-Egger regression analysis indicated no evidence of a pleiotropic effect (Egger intercept=3.302e-04, P=0.938). The leave-one-out sensitivity analyses and funnel plot further supported the stable, non-SNP-driven nature of the observed non-association (**Supplementary Figures S3J**, **S4J**).

### 3.4 Results of mediation analysis

Given the pivotal role of sex hormone-related phenotypes in addressing elevated SUA and female infertility, particularly the potential protective mediation of elevated SHBG, it becomes essential to comprehensively elucidate the impact of sex hormone-related phenotypes on female infertility. To achieve this, we conducted a two-step MR and bidirectional MR analyses, considering three sex hormone-related phenotypes. Our objective was to explore the mediating pathways from SUA levels to female infertility. In the initial step, a genetic instrument for SUA levels was utilized to estimate the causal effect on potential mediators. In the forward MR analysis, examining the relationship between SUA levels (as an exposure factor), sex hormone-related phenotypes (as mediators), and female infertility (as an outcome factor), we identified unidirectional causality between SUA and SHBG. Elevated SUA levels were significantly correlated with decreased levels of SHBG (β=-0.261; 95%CI: -0.399 to -0.123; P=2.177e-04). In the subsequent step, we assessed the causal effect of SHBG on the risk of female infertility using genetic tools for SHBG. We found evidence supporting a causal effect of SHBG on the risk of female infertility (OR=0.854; 95% CI, 0.793–0.920; P=2.853e-05). Calculating the indirect effect of SUA levels on female infertility through SHBG, we found a mediated effect of 0.041 (95%CI, 0.015 to 0.074; P=0.006), with a mediated proportion of 27.93% (95%CI,0.302%, 0.310%). This resulted in a direct effect of SUA on female infertility (β=0.106).

Furthermore, in a parallel mediated MR investigation, we uncovered a substantial impact of TT levels on female infertility, OR=0.733 (95% CI, 0.562–0.955, P = 0.022). During our inverse MR analysis, probing the intricate interplay among the three sex hormone-related phenotypes and SUA, a distinct unidirectional causality surfaced between TT levels and SUA levels. Elevated TT levels were associated with decreased SUA levels (β=-0.127; 95%CI: -0.225 to -0.030; P=0.010). Validation of the previously employed genetic instrument for SUA reaffirmed the causal role of SUA as a mediator influencing susceptibility to female infertility at this juncture. Finally, we estimated the indirect effect of TT levels on female infertility through SUA levels, revealing a mediated effect of β = -0.0187 (95% CI, -0.041 to -0.003; P = 0.046, calculated using the Goodman test equation), with the proportion of mediators being 6.01% (95% CI, 0.066%, 0.071%). This culminated in a direct effect of TT level on female infertility (β= -0.293).

## 4 Discussion

Female infertility is a condition with a complex etiology, influenced by multiple biological mechanisms and pathways (7, 8, 27). Our study, utilizing a large genome-wide association dataset, employed two-sample and mediated Mendelian Randomization analyses to explore potential causal links between SUA, sex hormone-related phenotypes, and female infertility. Our findings strongly support the mediation of hazardous causal effects of SUA on female infertility risk by SHBG, with no evidence of a reverse causal effect. Consistent with recent observational epidemiological literature, these studies delve into multiple mechanisms underlying the association between uric acid and female reproductive disorders (28–31). Chronic high uric acid levels are linked to insulin resistance, abnormal lipid metabolism, and complications associated with PCOS, including abnormal sex hormone levels (7, 14, 32). Moreover, uric acid may contribute to endometriosis through pro-inflammatory pathways involving interleukin-1β and the NOD-like receptor protein inflammasome (33). The involvement of uric acid in inflammation and oxidative stress, including lipid oxidation in adipocytes, further emphasizes its role in disrupting reproductive hormone levels and influencing endothelial dysfunction (34, 35).Our focus on plasma SHBG reveals its potential role as a mediator in the observed relationships. Hepatocyte nuclear factor-4α (HNF-4α), a key transcription factor in SHBG synthesis, regulates hepatic SHBG levels, influencing glucose and lipid metabolism (36, 37). Low serum SHBG levels, associated with metabolic abnormalities and insulin resistance, are detected not only in the liver but also in various tissues of the female reproductive tract (38). Lower SHBG concentrations are predictive of hyperuricemia, suggesting a potential link between SUA and SHBG production via AMPK inactivation in the liver (39).

In investigating the relationship between TT and uric acid, our findings align with some observational studies (40, 41). Wan et al. (42) and Han et al. (40) reported negative correlations between SUA levels and TT, while Feldman et al. (43) found that asymptomatic hyperuricemia was associated with lower TT levels. Mechanistically, we propose that low testosterone levels may lead to insulin resistance, affecting SUA clearance (44, 45), and sex hormones may influence SUA levels by modulating renal urate excretion (46). Concerning the causal interpretation of TT and female infertility, the literature presents conflicting views (47, 48). Some studies propose androgens as high-risk factors for female infertility, particularly in PCOS patients (49), while others find no association with live births or pregnancies (50). The complexity arises from varying methodologies and the absence of prospective randomized trials. The potential role of androgen deficiency or excess is explored in the context of ovarian reserve function, considering the benefits of Dehydroepiandrosterone (DHEA) supplementation in hypoandrogenic women with low ovarian reserve function (51, 52). The regulation of ovarian function by androgens, both in hyperandrogenism associated with PCOS and hypoandrogenism in low ovarian reserve, underscores the importance of androgens in fertility (53). Associations between androgen treatment and improved outcomes in endometrial diseases, such as endometriosis, are explored, emphasizing the potential role of androgens in endometrial health (54, 55). While debates persist in the literature, selective androgens are proposed as potential pregnancy-promoting drugs (56). This comprehensive view helps to illuminate the intricate interplay between serum uric acid, sex hormones, and female infertility, highlighting potential biological pathways and mediators that could be targeted in future research and clinical interventions.

While our study elucidates the complex interplay between serum uric acid, sex hormones, and female infertility, it’s important to acknowledge its limitations. The use of Mendelian Randomization helped minimize confounding, but the biological roles of our genetic tools and the lack of comprehensive GWAS data on certain variables, including medication use and inflammatory factors, might limit the interpretation and generalizability of our findings. Our analysis mainly reflects data from European populations, highlighting a need for broader demographic inclusivity in future studies. Future research should expand to include diverse populations and additional confounding factors such as drug effects and inflammation to enhance our understanding and the clinical applicability of these relationships.

## 5 Conclusions

Our study, utilizing GWAS, two-sample analyses, and mediated MR, has illuminated complex relationships between SUA levels, sex hormone-related phenotypes, and female infertility. We identified a significant positive correlation between elevated SUA levels and an increased risk of infertility in women, with SHBG partially mediating this association. This discovery suggests the potential role of SUA and SHBG as biomarkers for assessing female infertility risk, although further research is needed to validate these findings. Additionally, our results shed light on the role of TT in female infertility, suggesting its indirect influence on reproductive function via modulation of SUA levels. This novel insight provides a basis for exploring new strategies to improve female reproductive health and address infertility challenges. However, the study’s conclusions warrant careful interpretation. The associations we identified, particularly between SUA and sex hormone-related phenotypes, call for further validation through larger, more comprehensive studies. Future research should aim to gather more extensive data to confirm these findings and explore the underlying physiological mechanisms of female infertility in greater depth.

## Data availability statement

The original contributions presented in the study are included in the article/**Supplementary Material**. Further inquiries can be directed to the corresponding author.

## Ethics statement

This study only used published or publicly available data. Ethical approval for each study included in the investigation can be found in the original publications (including informed consent from each participant).

## Author contributions

ZT: Conceptualization, Data curation, Formal analysis, Investigation, Methodology, Project administration, Resources, Software, Visualization, Writing – original draft, Writing – review & editing. JS: Conceptualization, Data curation, Formal analysis, Investigation, Methodology, Project administration, Supervision, Validation, Visualization, Writing – original draft, Writing – review & editing. YH: Conceptualization, Data curation, Formal analysis, Investigation, Methodology, Project administration, Visualization, Writing – original draft. JL: Conceptualization, Data curation, Investigation, Methodology, Visualization, Writing – original draft. MD: Conceptualization, Data curation, Formal analysis, Investigation, Methodology, Project administration, Writing – original draft. AS: Conceptualization, Data curation, Investigation, Methodology, Project administration, Visualization, Writing – original draft. JH: Conceptualization, Data curation, Formal analysis, Investigation, Methodology, Project administration, Visualization, Writing – original draft. YY: Conceptualization, Data curation, Formal analysis, Methodology, Project administration, Visualization, Writing – original draft. SH: Conceptualization, Data curation, Formal analysis, Investigation, Methodology, Visualization, Writing – original draft. XZ: Conceptualization, Data curation, Formal analysis, Investigation, Methodology, Validation, Visualization, Writing – original draft. RL: Formal analysis, Funding acquisition, Investigation, Project administration, Supervision, Writing – original draft, Writing – review & editing, Software, Data curation, Methodology, Resources, Visualization.
