# Supplementary material for: Decoding connections in the European population: serum uric acid, sex hormone-binding globulin, total testosterone, estradiol, and female infertility – advanced bidirectional and mediative Mendelian randomization
Source: Front Endocrinol (Lausanne). 2024 Jun 28;15:1398600. doi: 10.3389/fendo.2024.1398600 (PMC11239382; doi:10.3389/fendo.2024.1398600)
Supplement: Supplementary file 1 [file DataSheet_1.pdf]

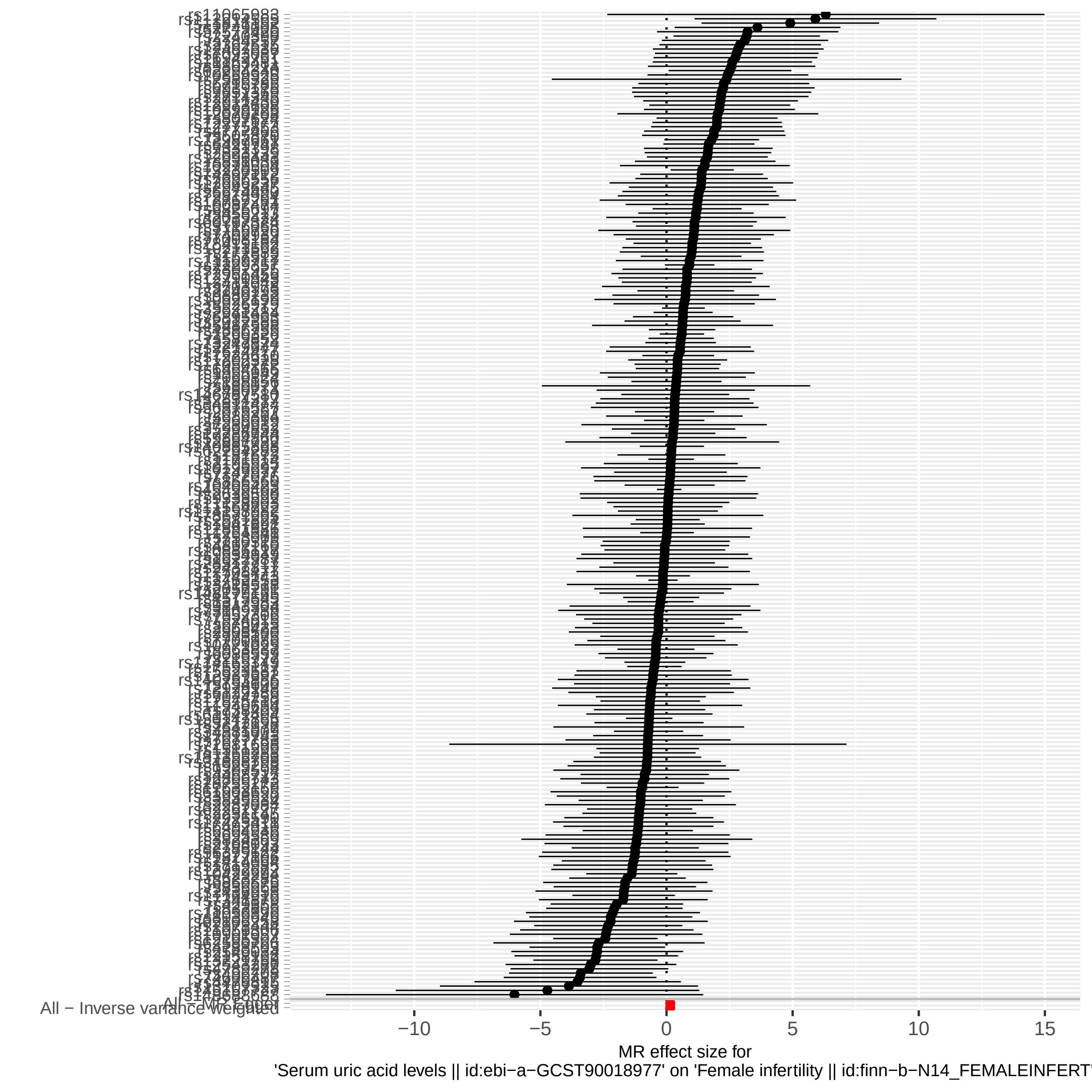

rs11065983
All − Inverse variance weighted
−10
−5
0
5
10
15
MR effect size for
'Serum uric acid levels || id:ebi−a−GCST90018977' on 'Female infertility || id:finn−b−N14_FEMALEINFERT

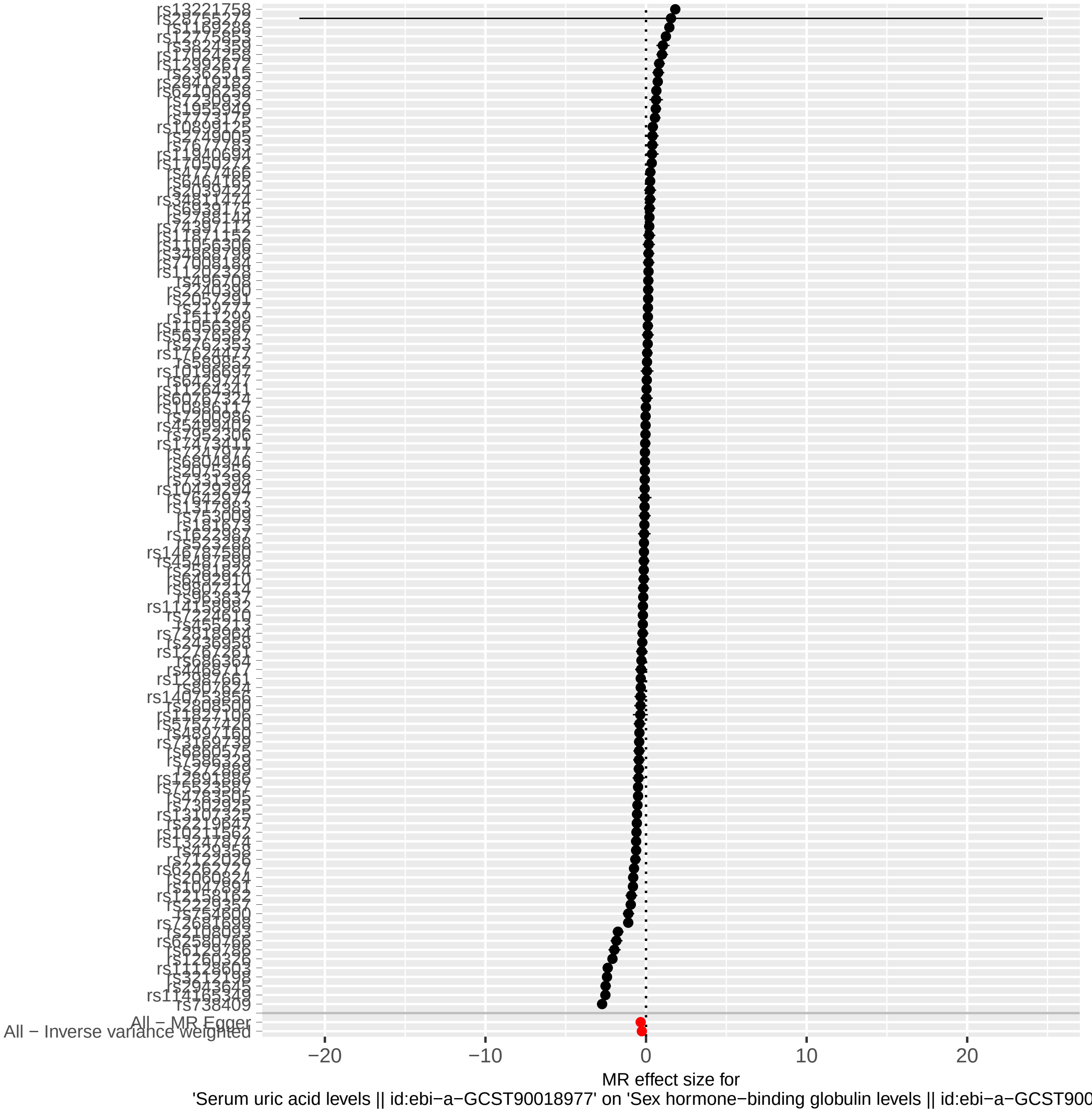

rs13221758
rs738409
All − MR Egger
All − Inverse variance weighted
−20
−10
0
10
20
MR effect size for
'Serum uric acid levels || id:ebi−a−GCST90018977' on 'Sex hormone−binding globulin levels || id:ebi−a−GCST900

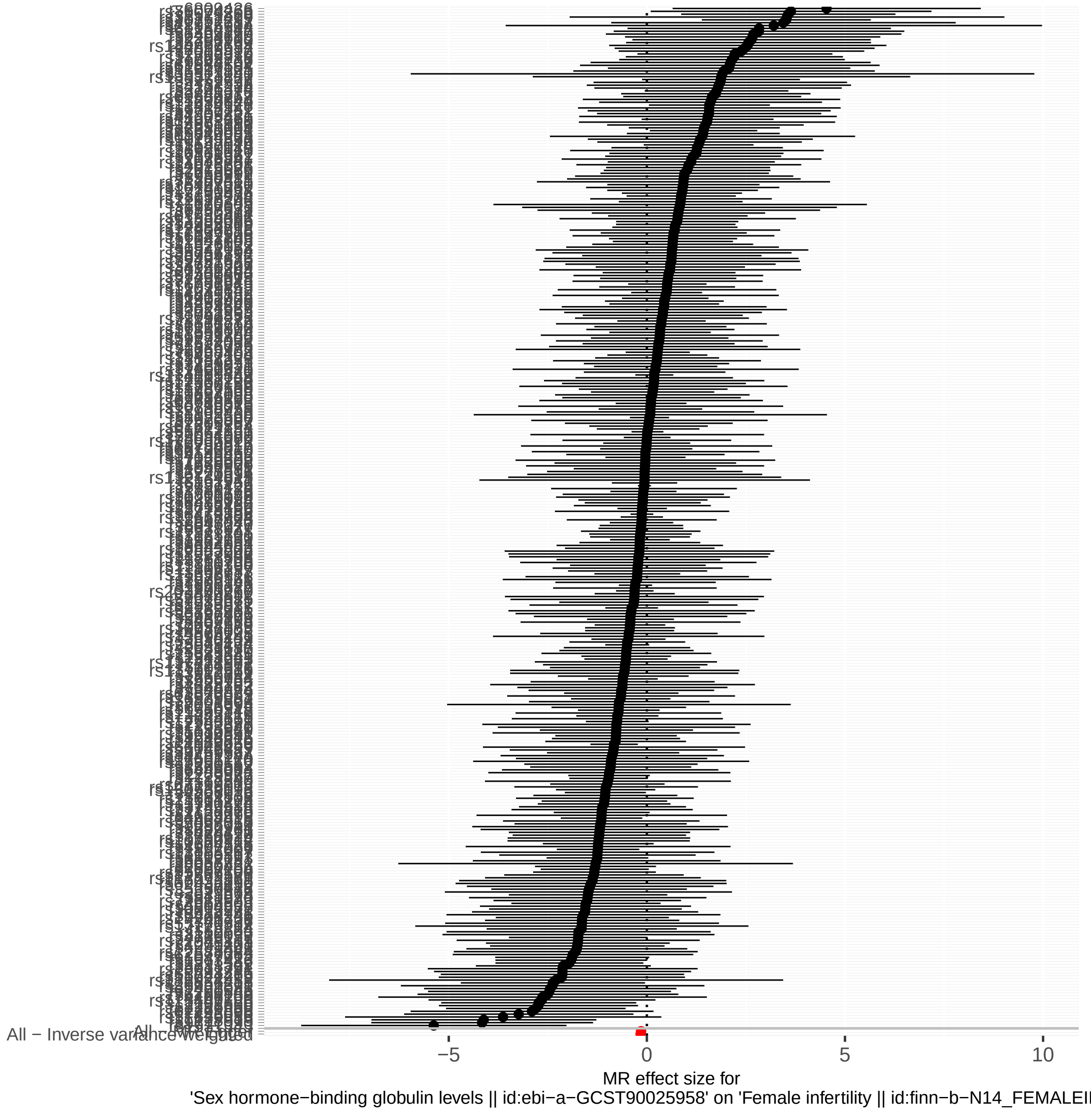
All − Inverse variance weighted
−5
0
5
10
MR effect size for
'Sex hormone−binding globulin levels || id:ebi−a−GCST90025958' on 'Female infertility || id:finn−b−N14_FEMALEI

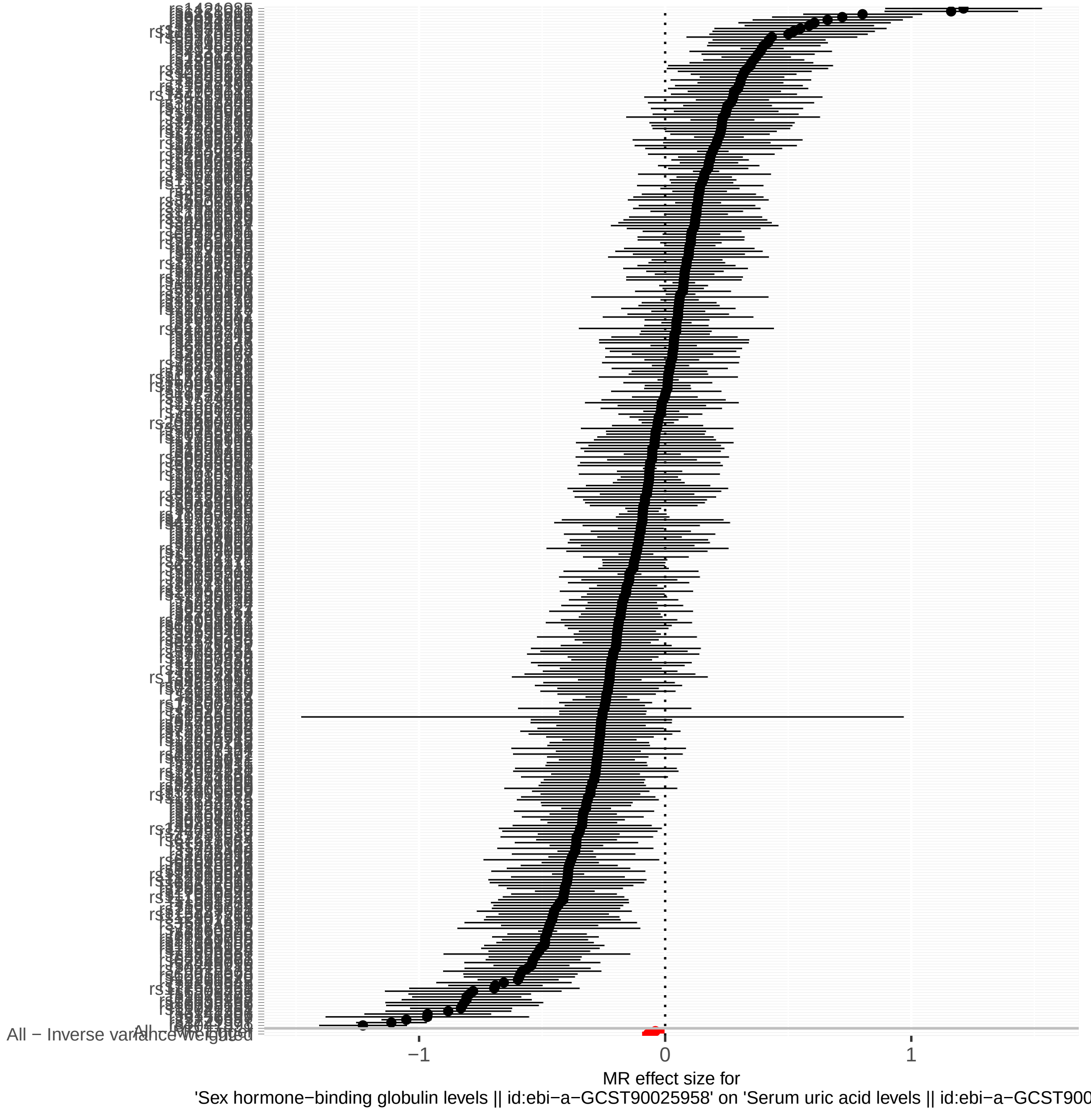

All − Inverse variance weighted
−1
0
1
MR effect size for
'Sex hormone−binding globulin levels || id:ebi−a−GCST90025958' on 'Serum uric acid levels || id:ebi−a−GCST900

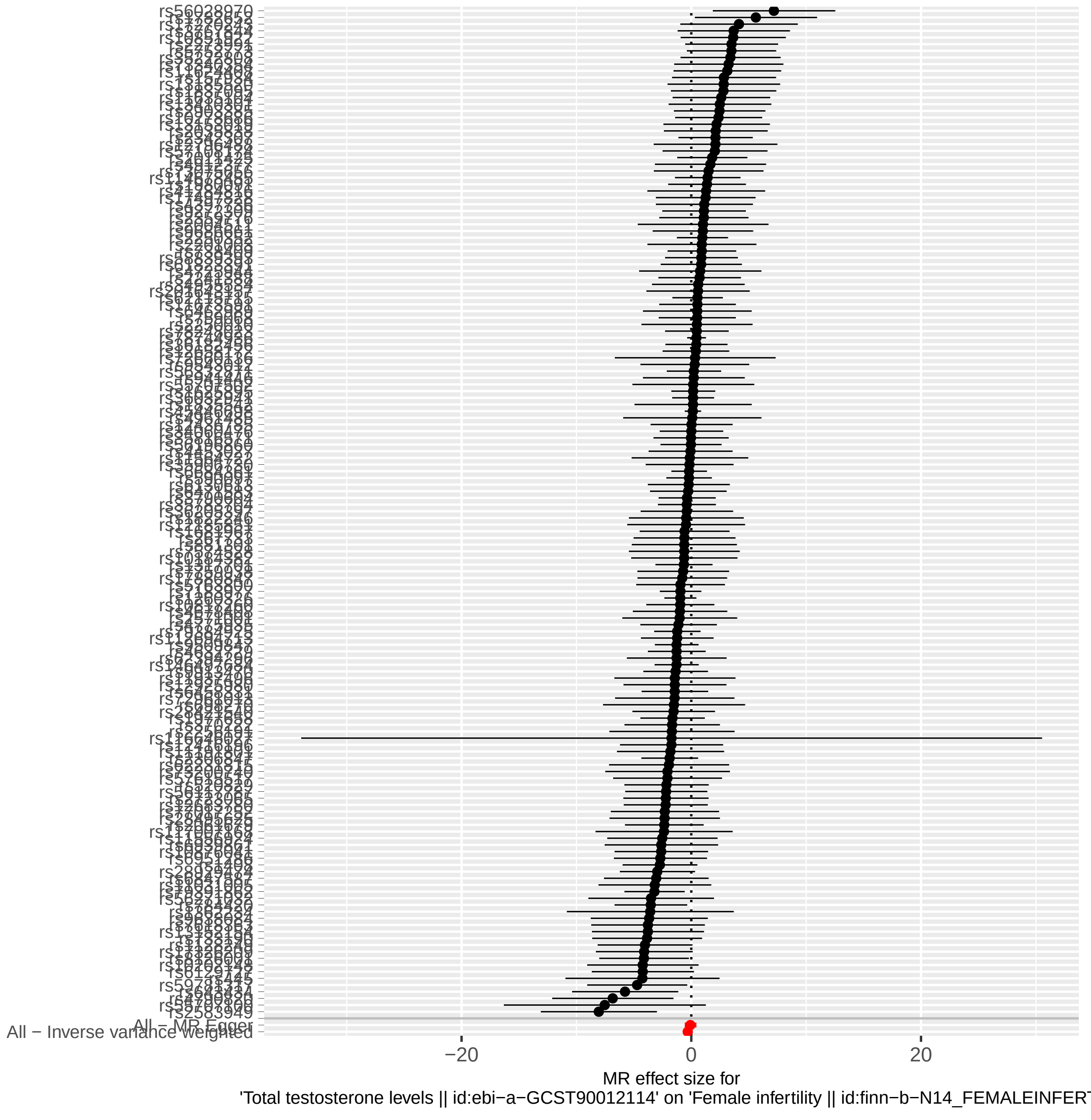
rs56028070
rs2583949
All − MR Egger
All − Inverse variance weighted
−20
0
20
MR effect size for
'Total testosterone levels || id:ebi−a−GCST90012114' on 'Female infertility || id:finn−b−N14_FEMALEINFER

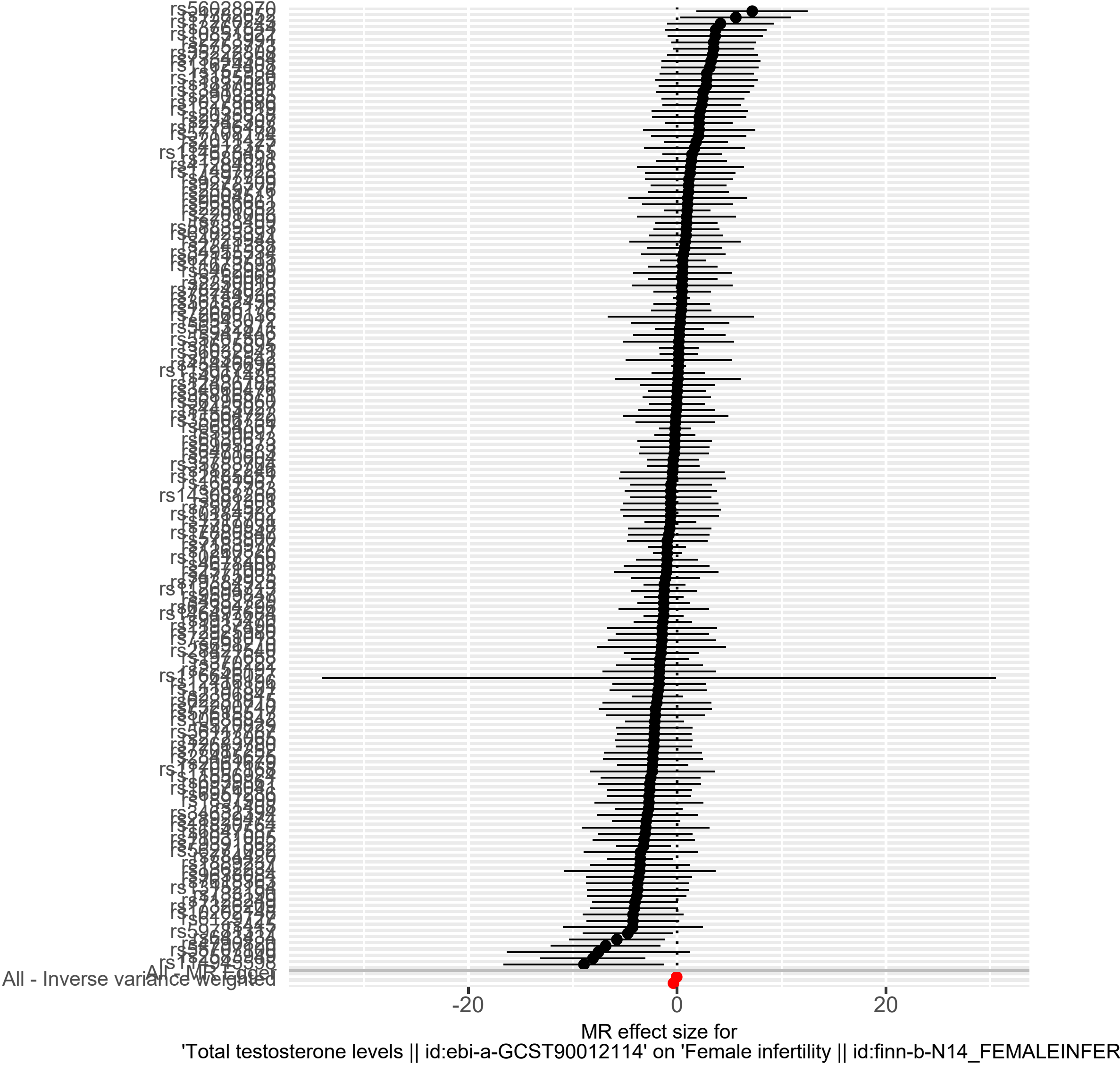
All - MR Egger
All - Inverse variance weighted
-20
0
20
MR effect size for
'Total testosterone levels || id:ebi-a-GCST90012114' on 'Female infertility || id:finn-b-N14_FEMALEINFER

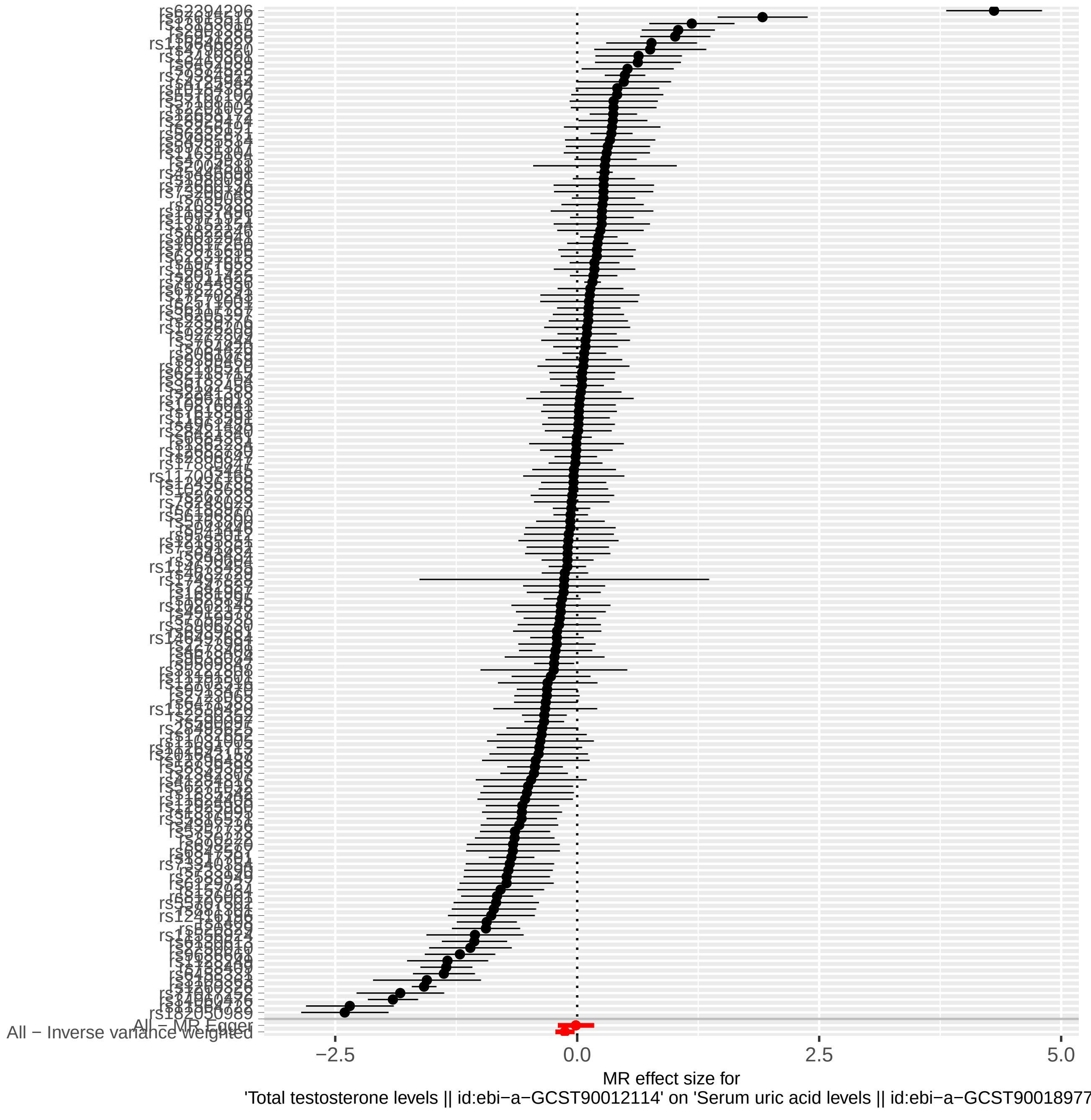
rs62294206
All − MR Egger
All − Inverse variance weighted
−2.5
0.0
2.5
5.0
MR effect size for
'Total testosterone levels || id:ebi−a−GCST90012114' on 'Serum uric acid levels || id:ebi−a−GCST90018977'

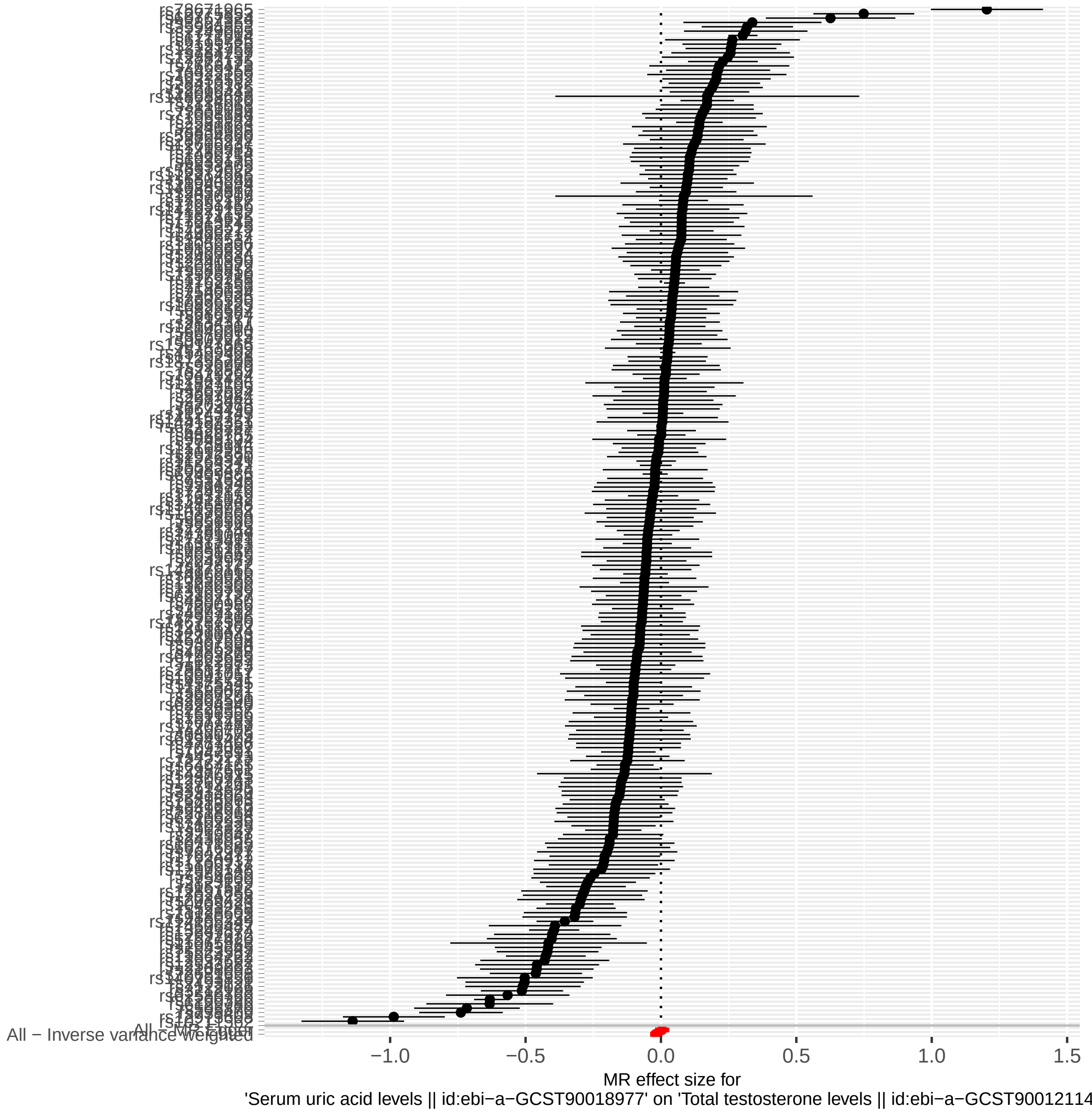

rs78671065
All − MR Egger
All − Inverse variance weighted
−1.0
−0.5
0.0
0.5
1.0
1.5
MR effect size for
'Serum uric acid levels || id:ebi−a−GCST90018977' on 'Total testosterone levels || id:ebi−a−GCST90012114

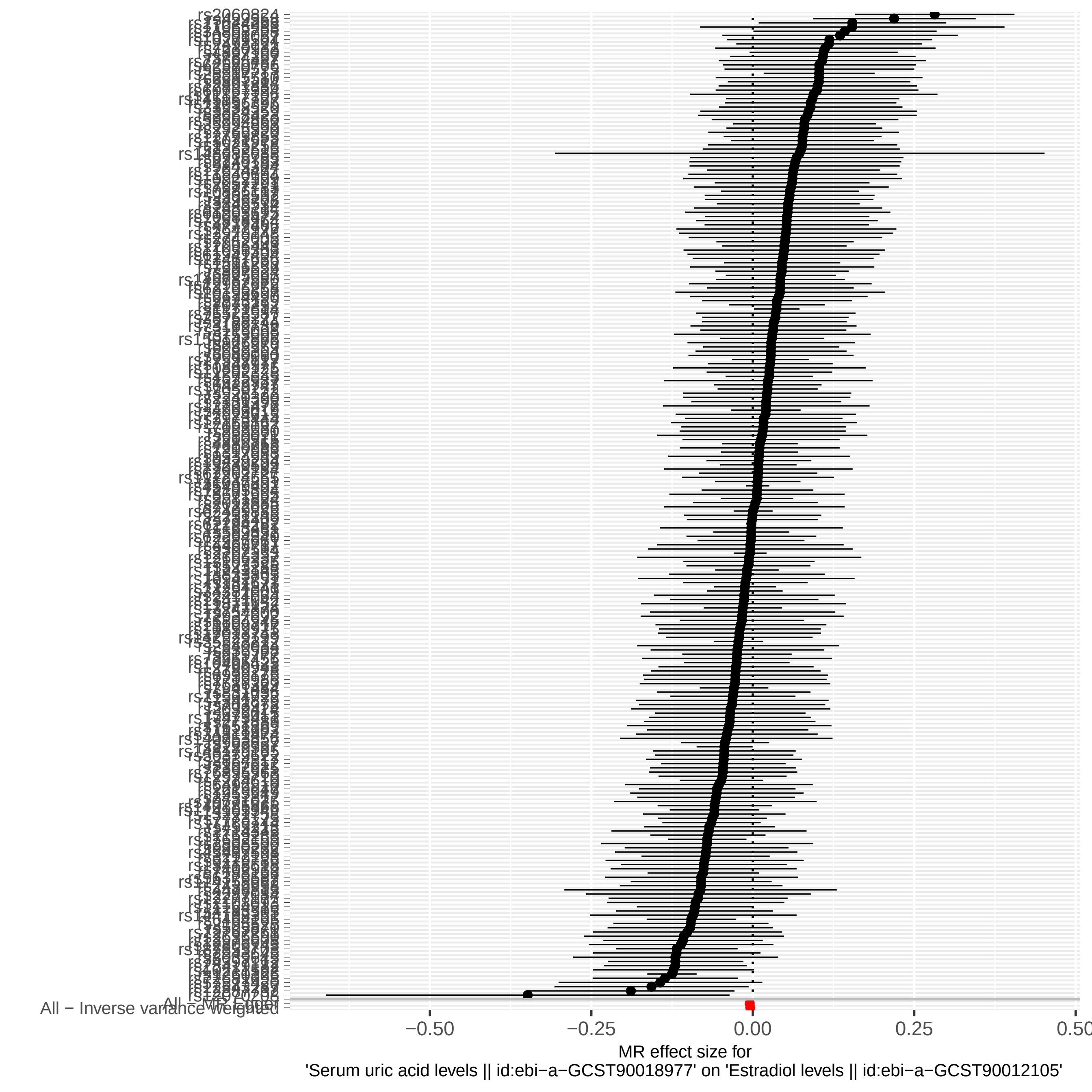
rs2060824
All − MR Egger
All − Inverse variance weighted
−0.50
−0.25
0.00
0.25
0.50
MR effect size for
'Serum uric acid levels || id:ebi−a−GCST90018977' on 'Estradiol levels || id:ebi−a−GCST90012105'

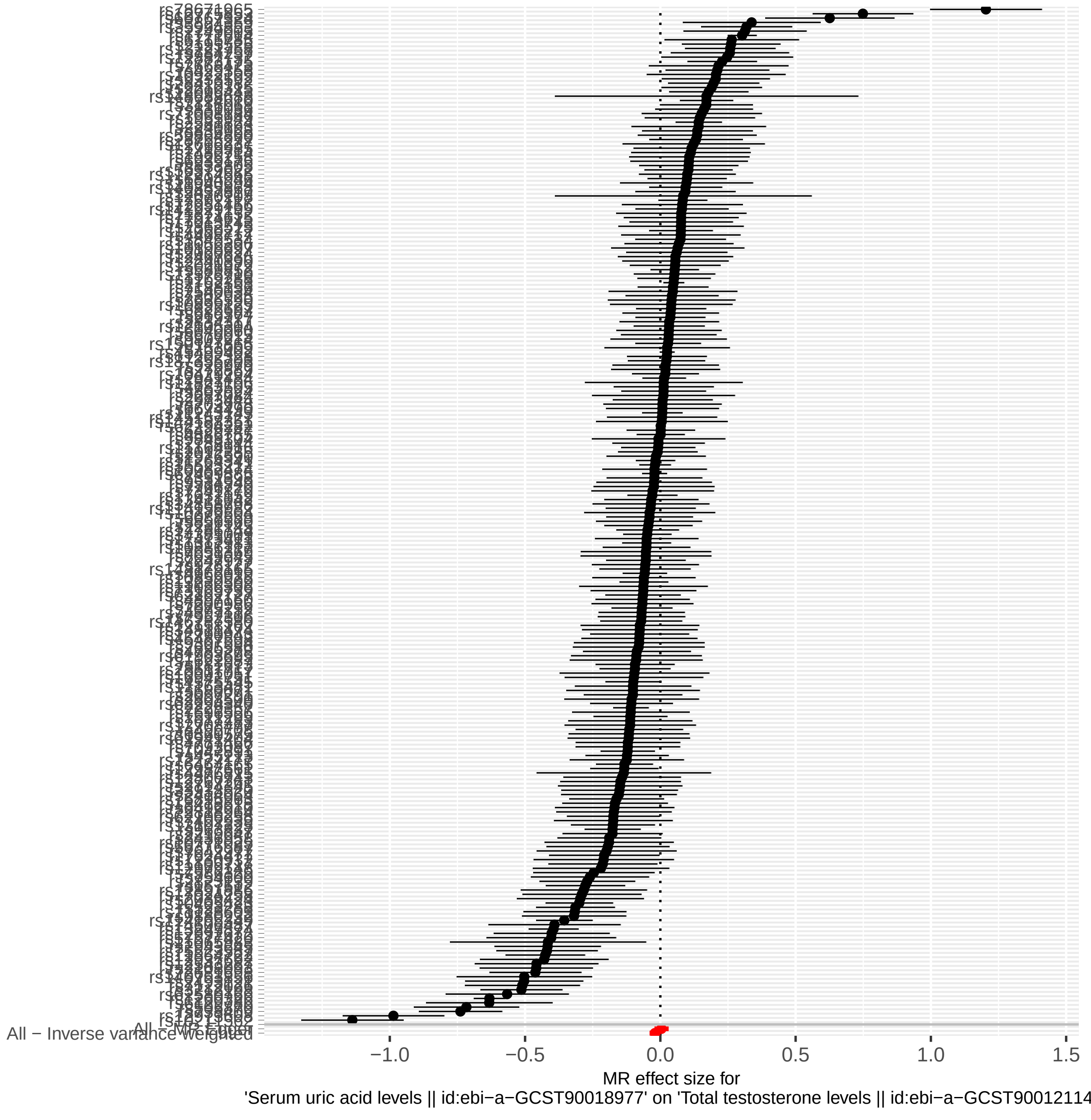

rs78671065
All − Inverse variance weighted
−1.0
−0.5
0.0
0.5
1.0
1.5
MR effect size for
'Serum uric acid levels || id:ebi−a−GCST90018977' on 'Total testosterone levels || id:ebi−a−GCST90012114'
