# Supplementary material for: Decoding connections in the European population: serum uric acid, sex hormone-binding globulin, total testosterone, estradiol, and female infertility – advanced bidirectional and mediative Mendelian randomization
Source: Front Endocrinol (Lausanne). 2024 Jun 28;15:1398600. doi: 10.3389/fendo.2024.1398600 (PMC11239382; doi:10.3389/fendo.2024.1398600)
Supplement: Supplementary file 2 [file DataSheet_2.pdf]

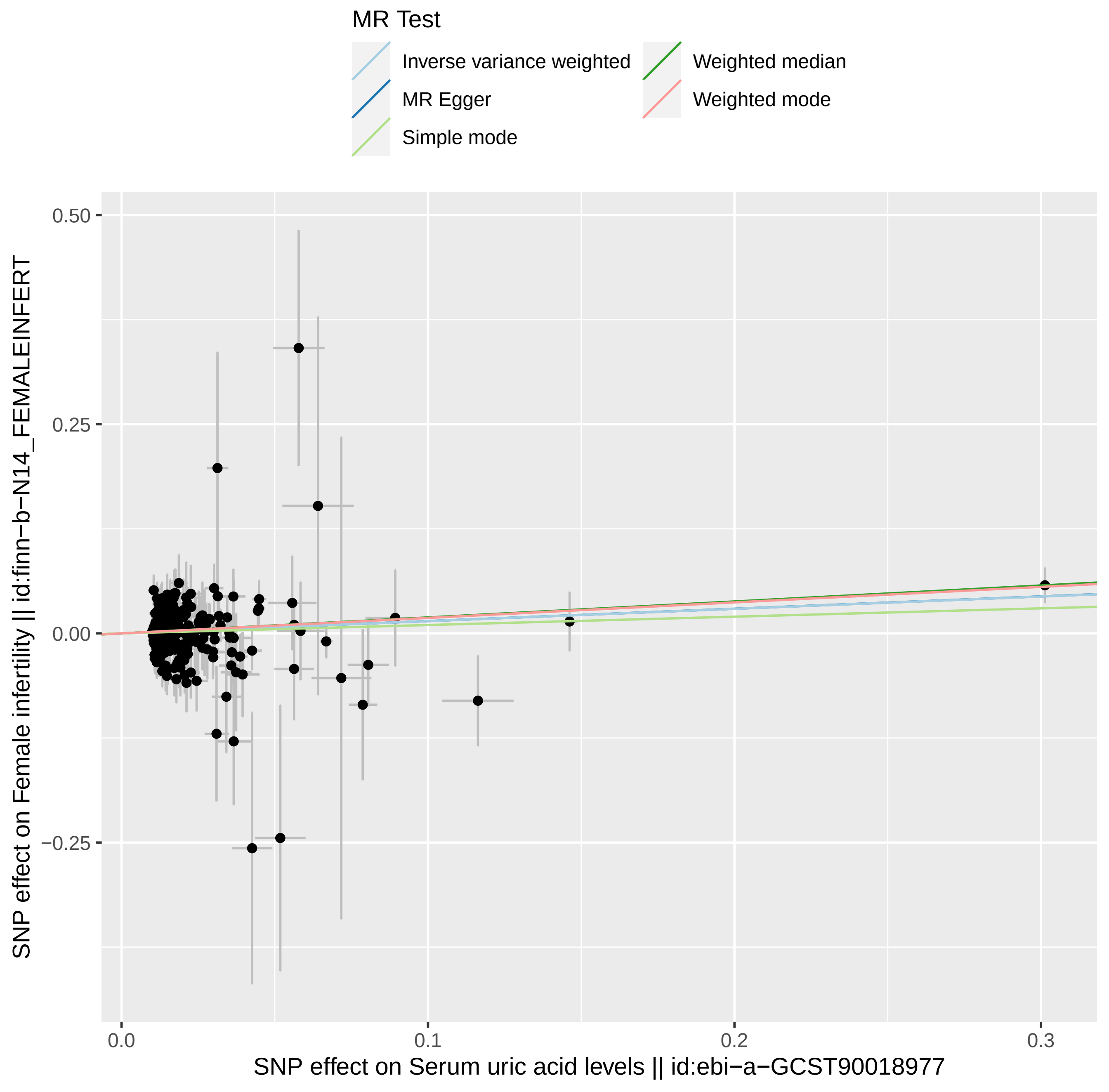
MR Test
Inverse variance weighted
MR Egger
Simple mode
Weighted median
Weighted mode
0.50
0.25
0.00
−0.25
0.0
0.1
0.2
0.3
SNP effect on Female infertility || id:finn−b−N14_FEMALEINFERT
SNP effect on Serum uric acid levels || id:ebi−a−GCST90018977

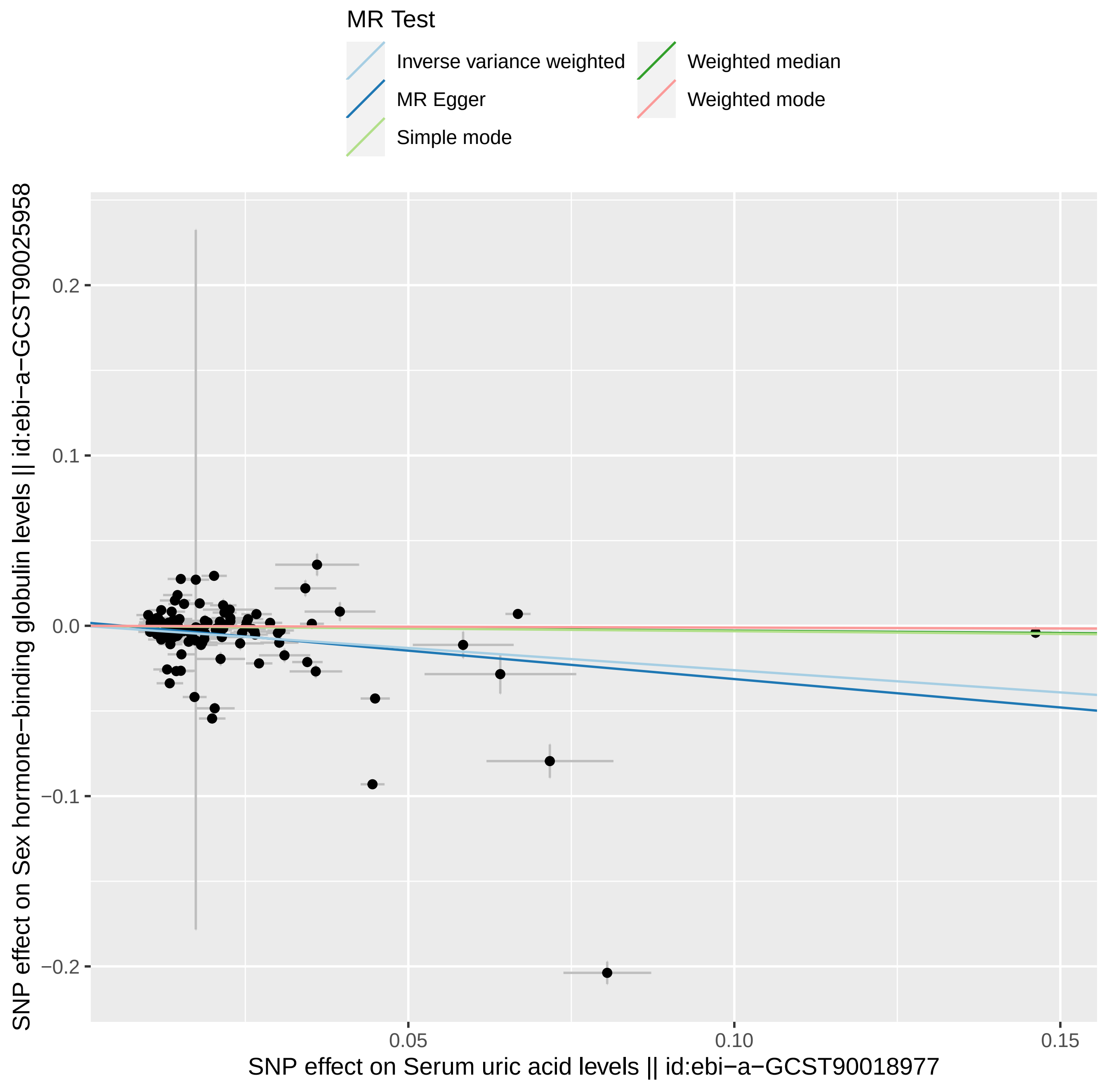
MR Test
Inverse variance weighted
MR Egger
Simple mode
Weighted median
Weighted mode
0.2
0.1
0.0
−0.1
−0.2
0.05
0.10
0.15
SNP effect on Sex hormone−binding globulin levels || id:ebi−a−GCST90025958
SNP effect on Serum uric acid levels || id:ebi−a−GCST90018977

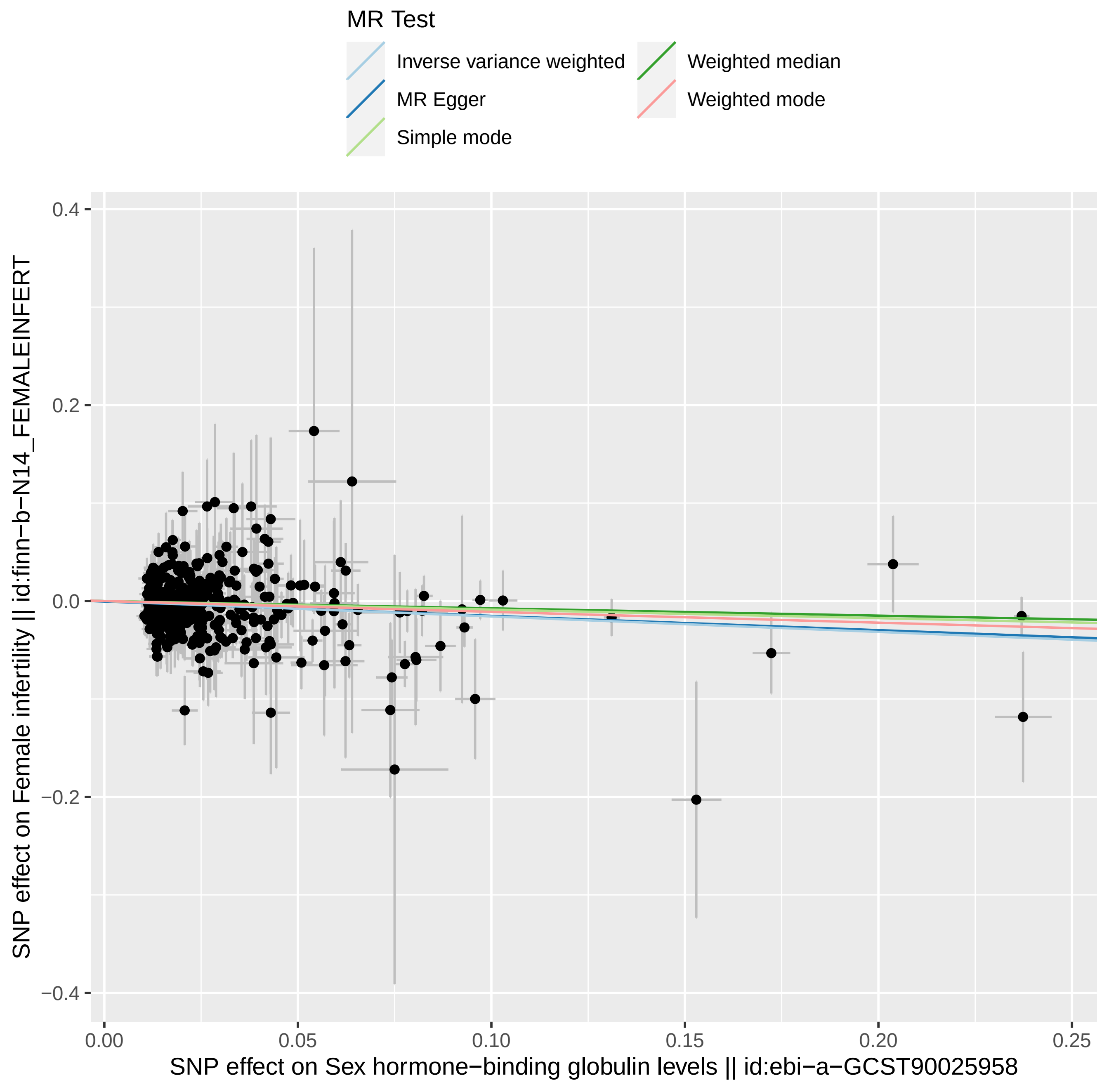
MR Test
Inverse variance weighted
MR Egger
Simple mode
Weighted median
Weighted mode
0.4
0.2
0.0
−0.2
−0.4
SNP effect on Female infertility || id:finn−b−N14_FEMALEINFERT
0.00
0.05
0.10
0.15
0.20
0.25
SNP effect on Sex hormone−binding globulin levels || id:ebi−a−GCST90025958

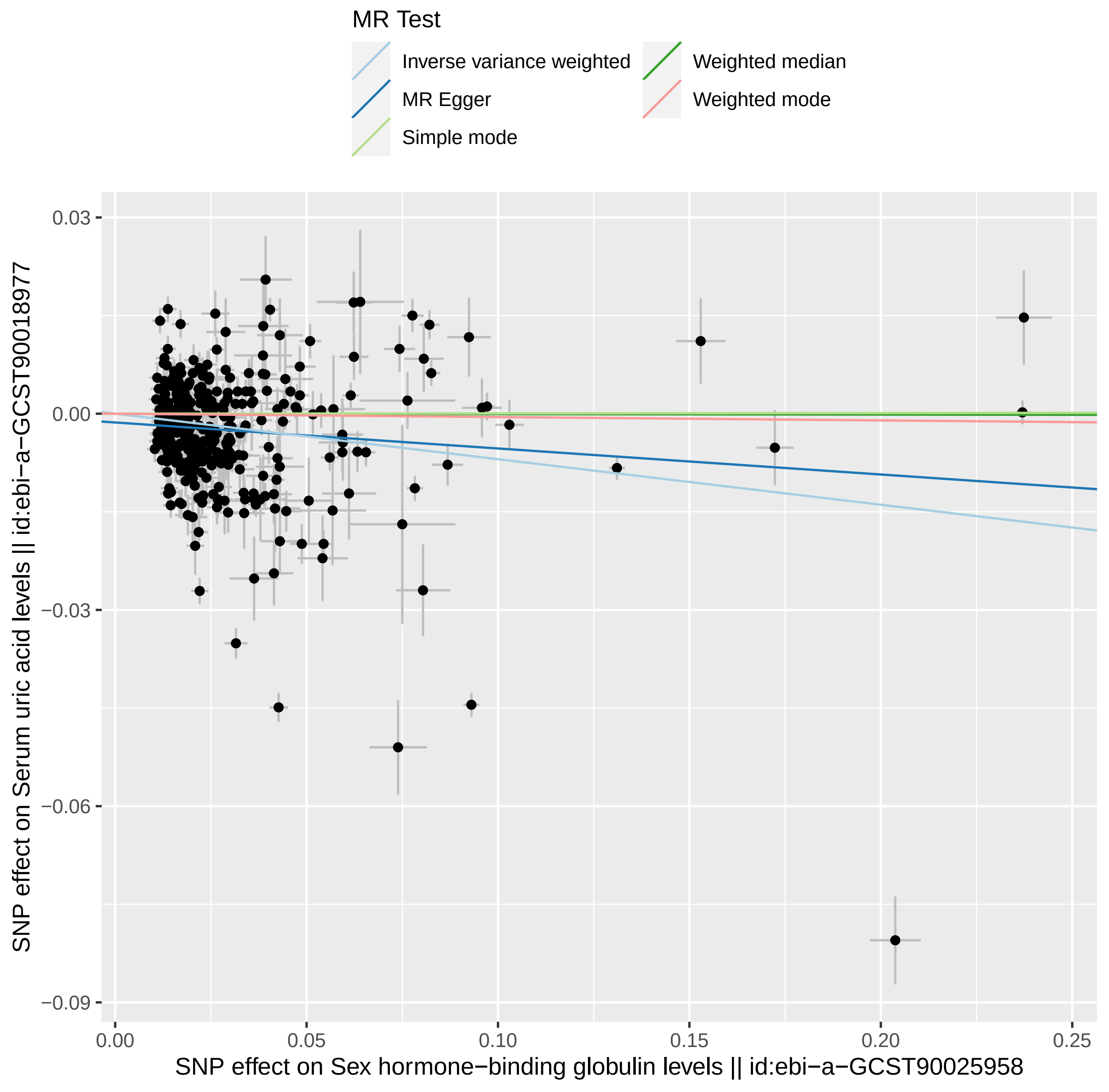
MR Test
Inverse variance weighted
MR Egger
Simple mode
Weighted median
Weighted mode
SNP effect on Serum uric acid levels || id:ebi−a−GCST90018977
0.03
0.00
−0.03
−0.06
−0.09
0.00
0.05
0.10
0.15
0.20
0.25
SNP effect on Sex hormone−binding globulin levels || id:ebi−a−GCST90025958

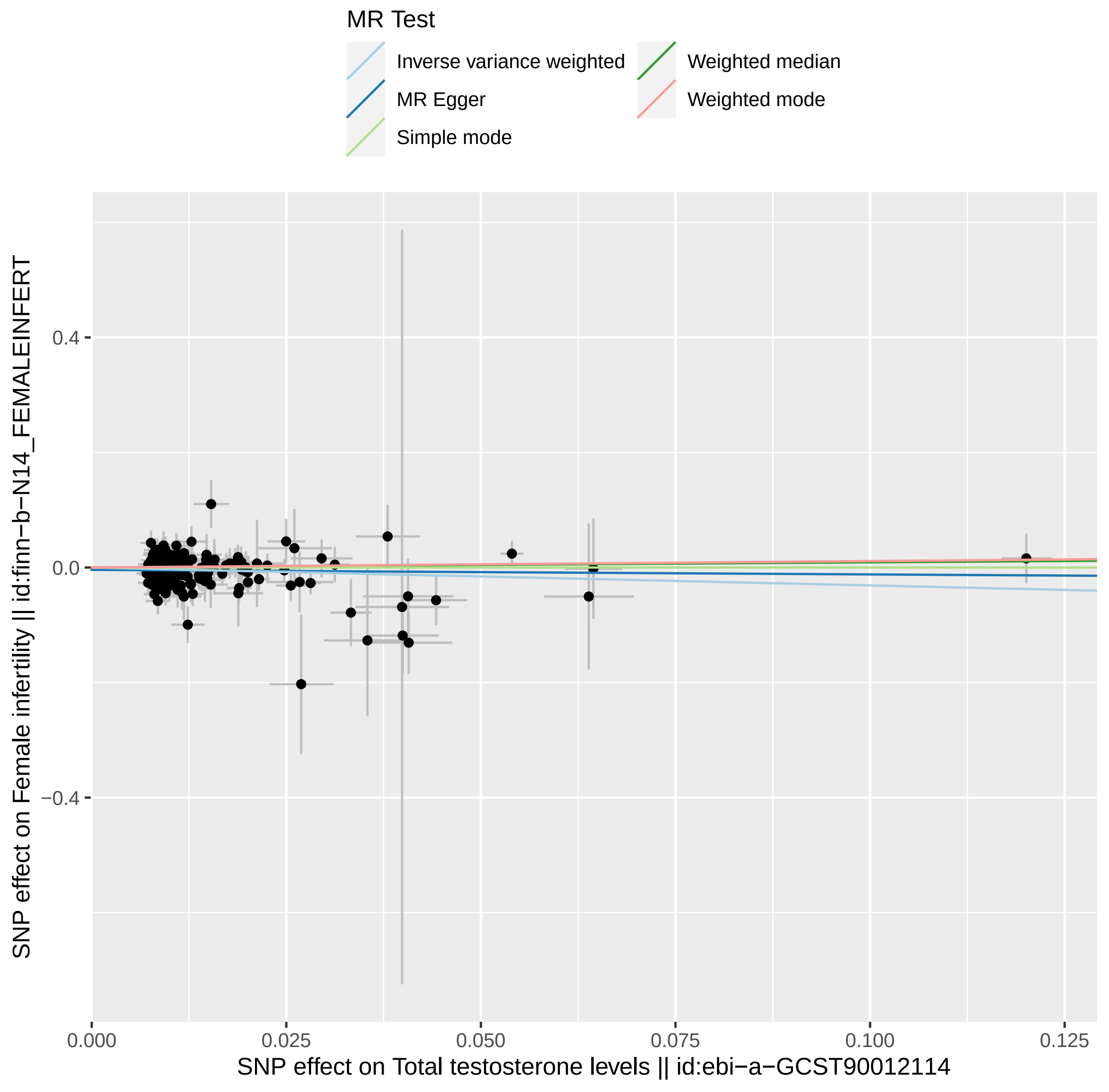
MR Test
Inverse variance weighted
MR Egger
Simple mode
Weighted median
Weighted mode
0.4
0.0
−0.4
SNP effect on Female infertility || id:finn−b−N14_FEMALEINFERT
0.000
0.025
0.050
0.075
0.100
0.125
SNP effect on Total testosterone levels || id:ebi−a−GCST90012114

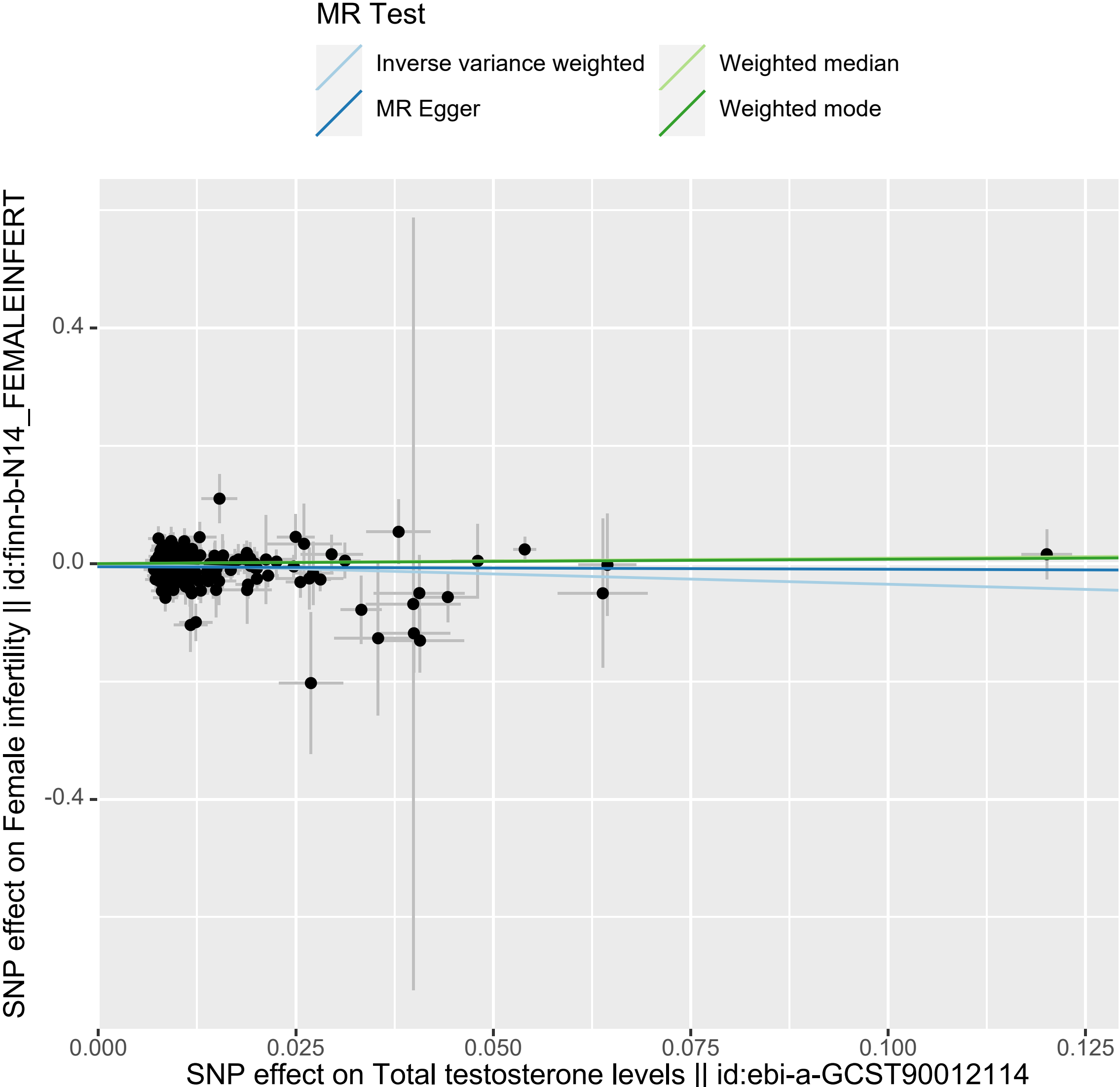

MR Test
Inverse variance weighted
MR Egger
Weighted median
Weighted mode
0.4
0.0
-0.4
0.000
0.025
0.050
0.075
0.100
0.125
SNP effect on Female infertility || id:finn-b-N14_FEMALEINFERT
SNP effect on Total testosterone levels || id:ebi-a-GCST90012114

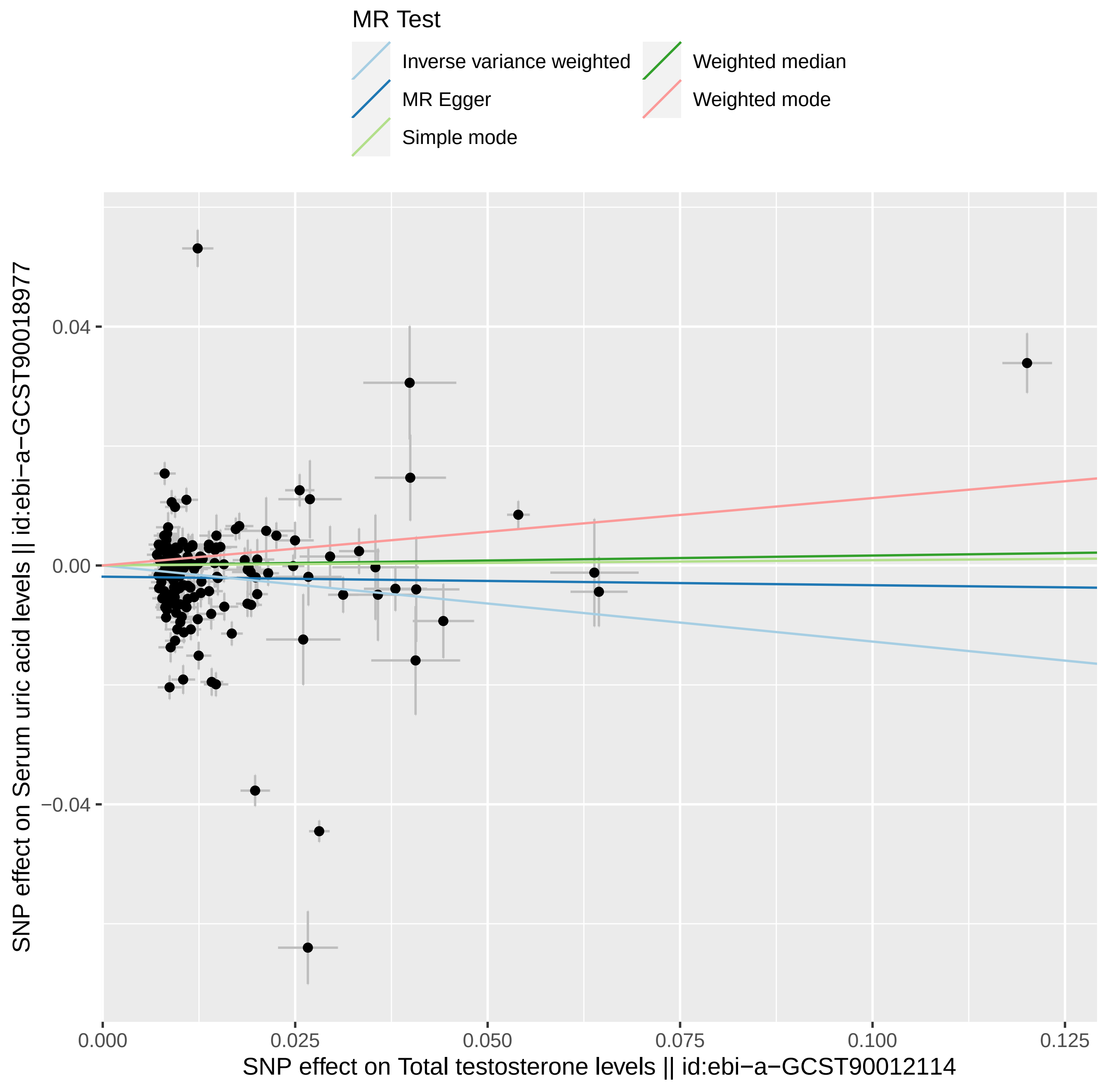

MR Test
Inverse variance weighted
MR Egger
Simple mode
Weighted median
Weighted mode
0.04
0.00
−0.04
SNP effect on Serum uric acid levels || id:ebi−a−GCST90018977
0.000
0.025
0.050
0.075
0.100
0.125
SNP effect on Total testosterone levels || id:ebi−a−GCST90012114

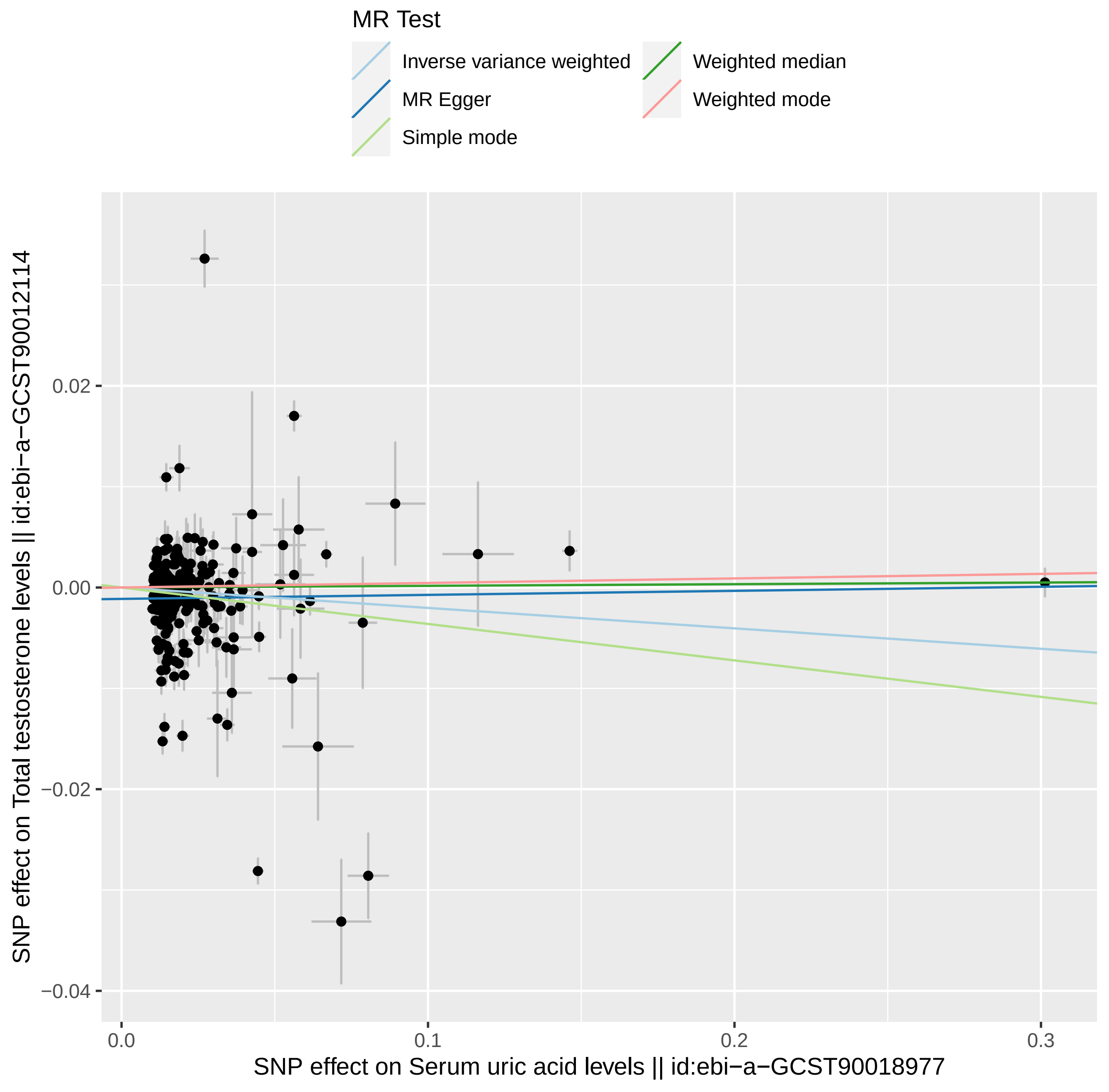
MR Test
Inverse variance weighted
MR Egger
Simple mode
Weighted median
Weighted mode
0.02
0.00
−0.02
−0.04
0.0
0.1
0.2
0.3
SNP effect on Total testosterone levels || id:ebi−a−GCST90012114
SNP effect on Serum uric acid levels || id:ebi−a−GCST90018977

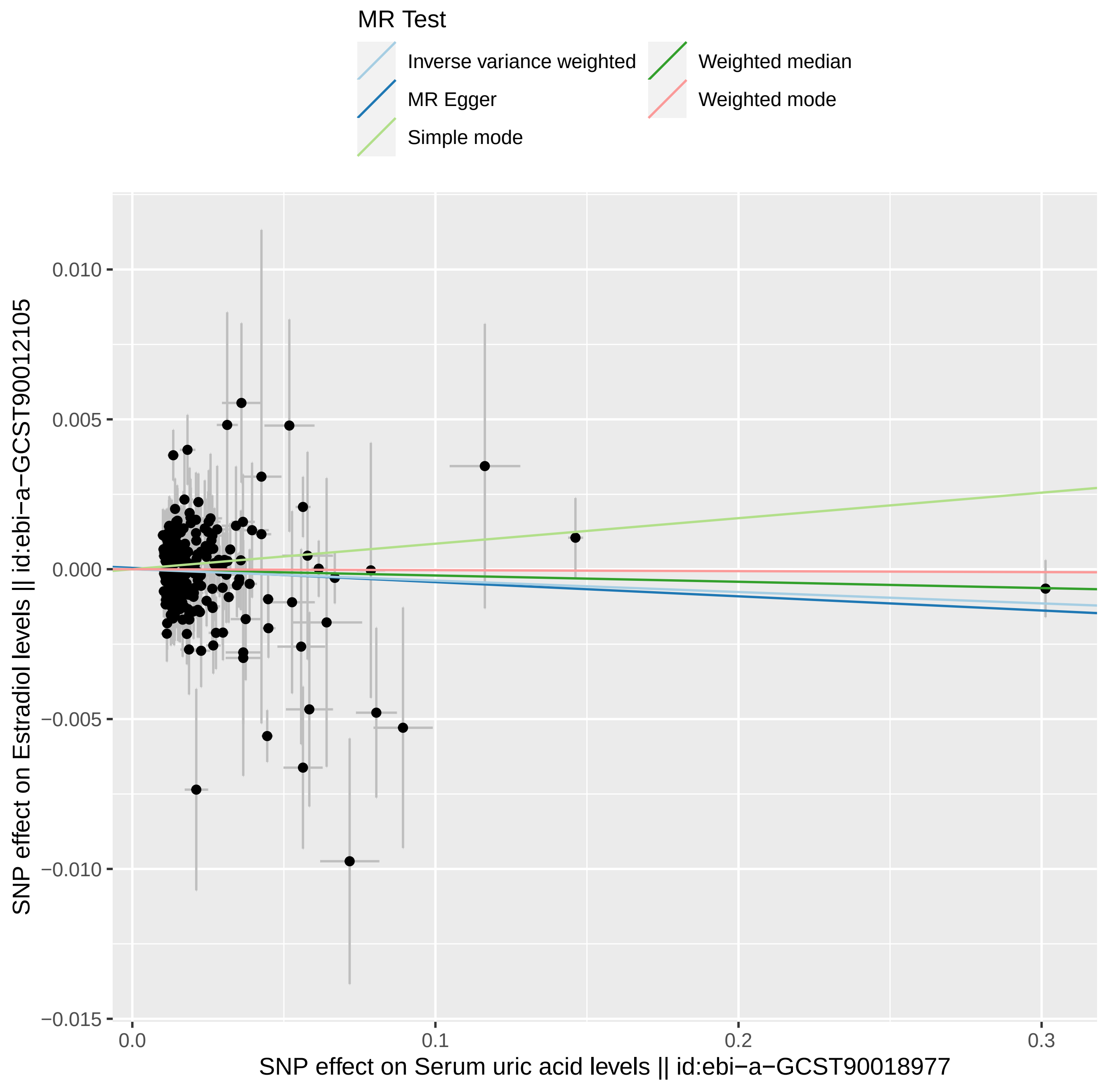
MR Test
Inverse variance weighted
MR Egger
Simple mode
Weighted median
Weighted mode
0.010
0.005
0.000
−0.005
−0.010
−0.015
0.0
0.1
0.2
0.3
SNP effect on Estradiol levels || id:ebi−a−GCST90012105
SNP effect on Serum uric acid levels || id:ebi−a−GCST90018977

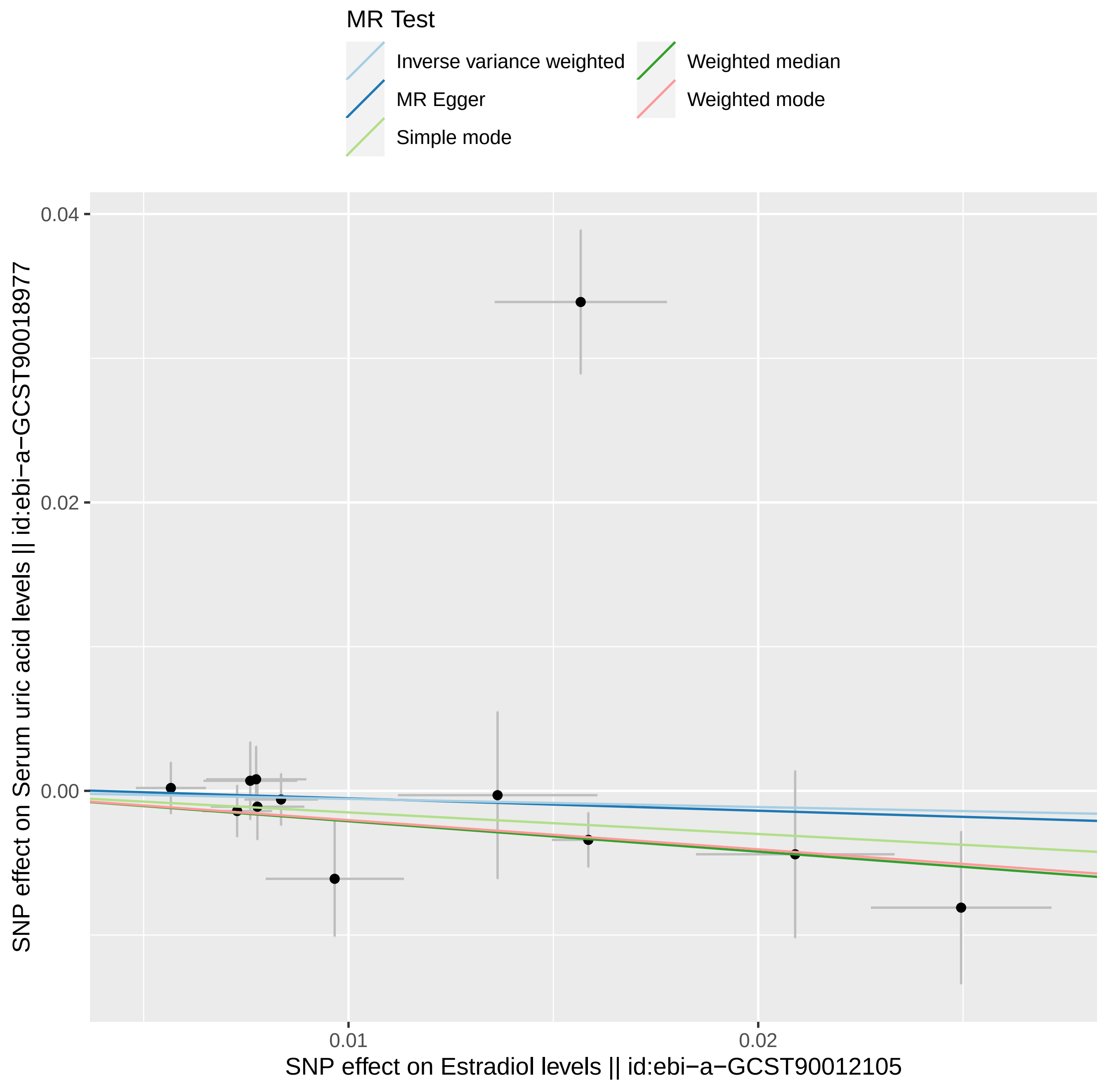
MR Test
Inverse variance weighted
MR Egger
Simple mode
Weighted median
Weighted mode
0.04
0.02
0.00
0.01
0.02
SNP effect on Serum uric acid levels || id:ebi−a−GCST90018977
SNP effect on Estradiol levels || id:ebi−a−GCST90012105
