# Supplementary material for: Decoding connections in the European population: serum uric acid, sex hormone-binding globulin, total testosterone, estradiol, and female infertility – advanced bidirectional and mediative Mendelian randomization
Source: Front Endocrinol (Lausanne). 2024 Jun 28;15:1398600. doi: 10.3389/fendo.2024.1398600 (PMC11239382; doi:10.3389/fendo.2024.1398600)
Supplement: Supplementary file 3 [file DataSheet_3.pdf]

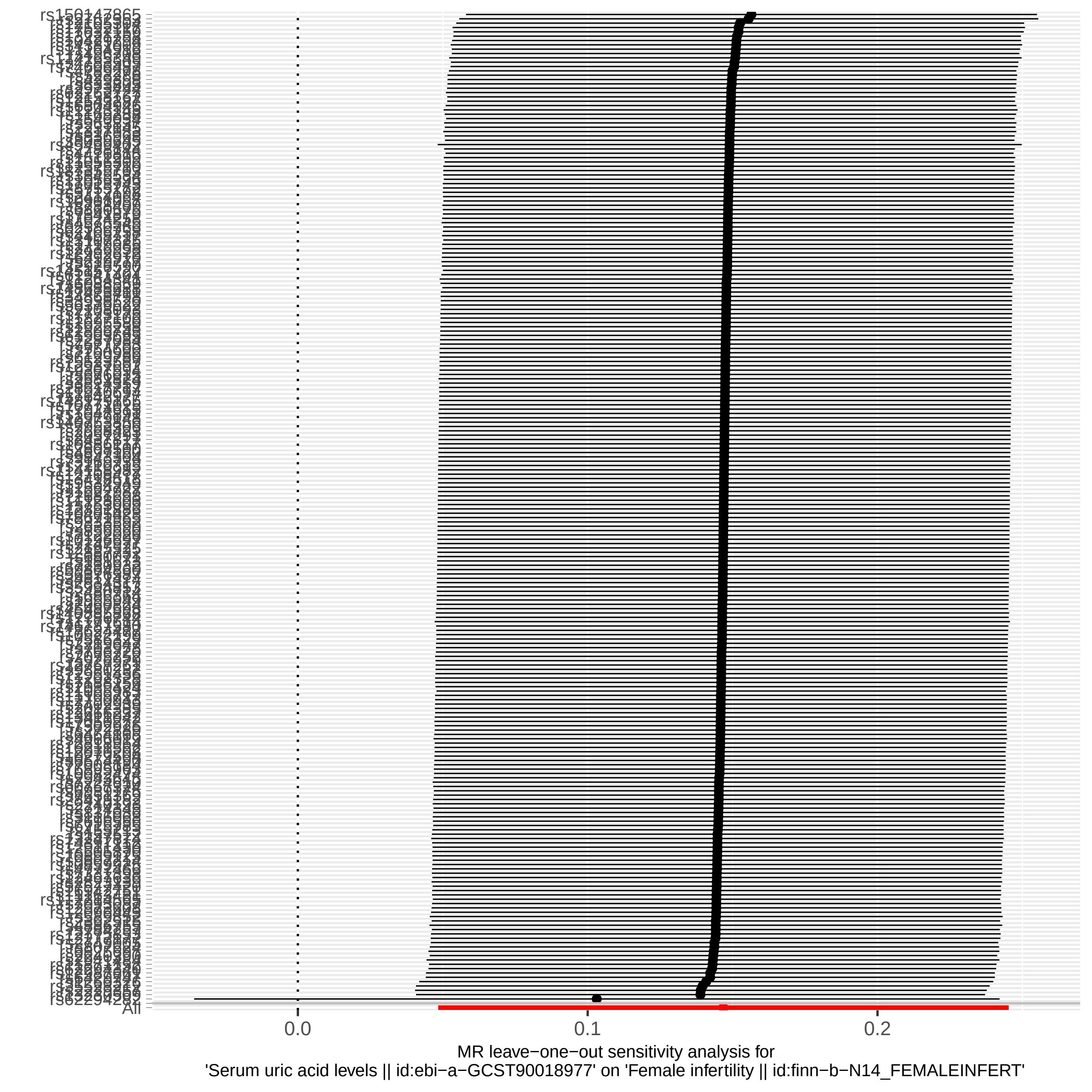
rs150147865
rs62294263
All
0.0
0.1
0.2
MR leave−one−out sensitivity analysis for
'Serum uric acid levels || id:ebi−a−GCST90018977' on 'Female infertility || id:finn−b−N14_FEMALEINFERT'

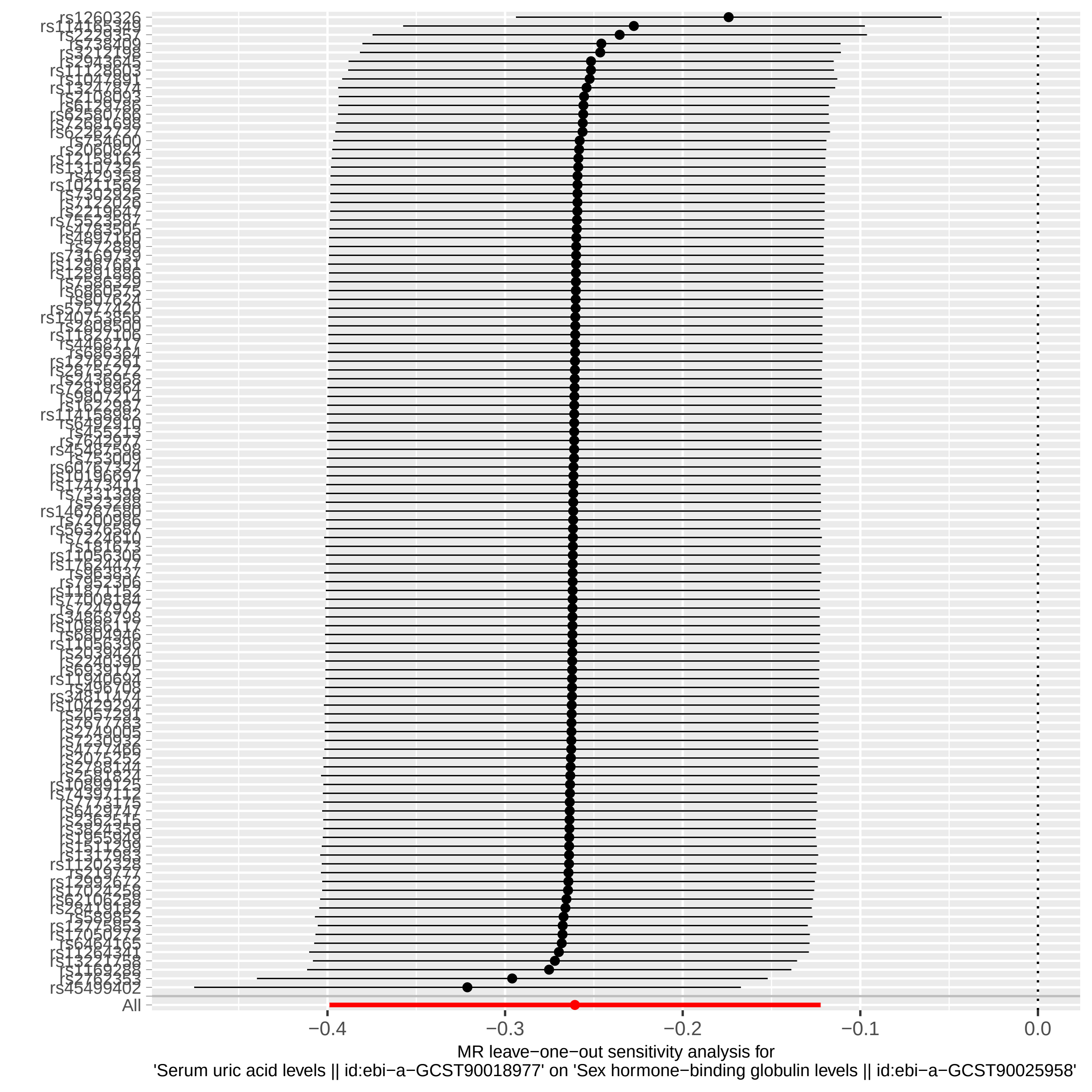
rs1260326
rs45499402
All
−0.4
−0.3
−0.2
−0.1
0.0
MR leave−one−out sensitivity analysis for
'Serum uric acid levels || id:ebi−a−GCST90018977' on 'Sex hormone−binding globulin levels || id:ebi−a−GCST90025958'

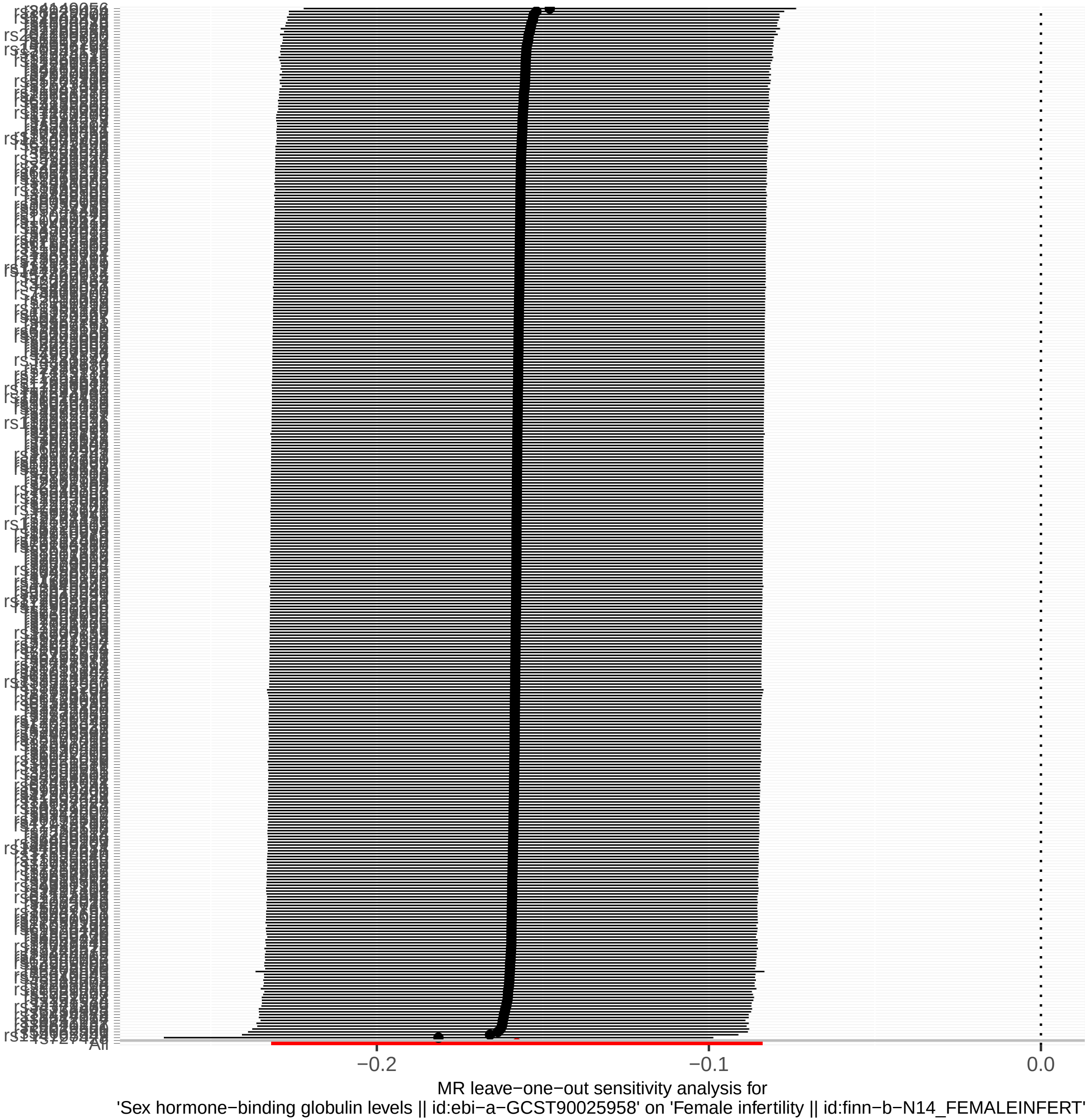
All
−0.2
−0.1
0.0
MR leave−one−out sensitivity analysis for
'Sex hormone−binding globulin levels || id:ebi−a−GCST90025958' on 'Female infertility || id:finn−b−N14_FEMALEINFERT'

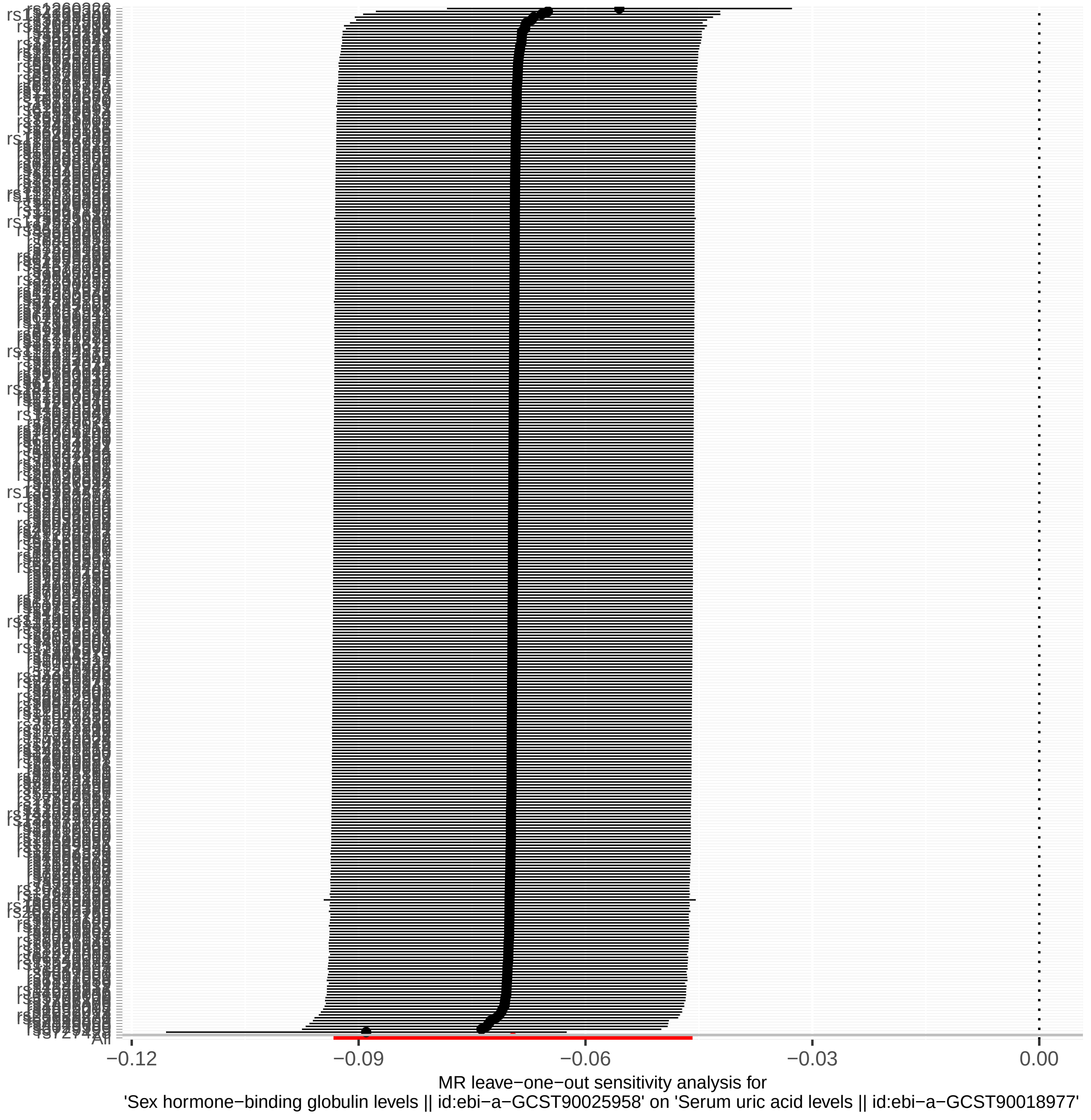
All
−0.12
−0.09
−0.06
−0.03
0.00
MR leave−one−out sensitivity analysis for
'Sex hormone−binding globulin levels || id:ebi−a−GCST90025958' on 'Serum uric acid levels || id:ebi−a−GCST90018977'

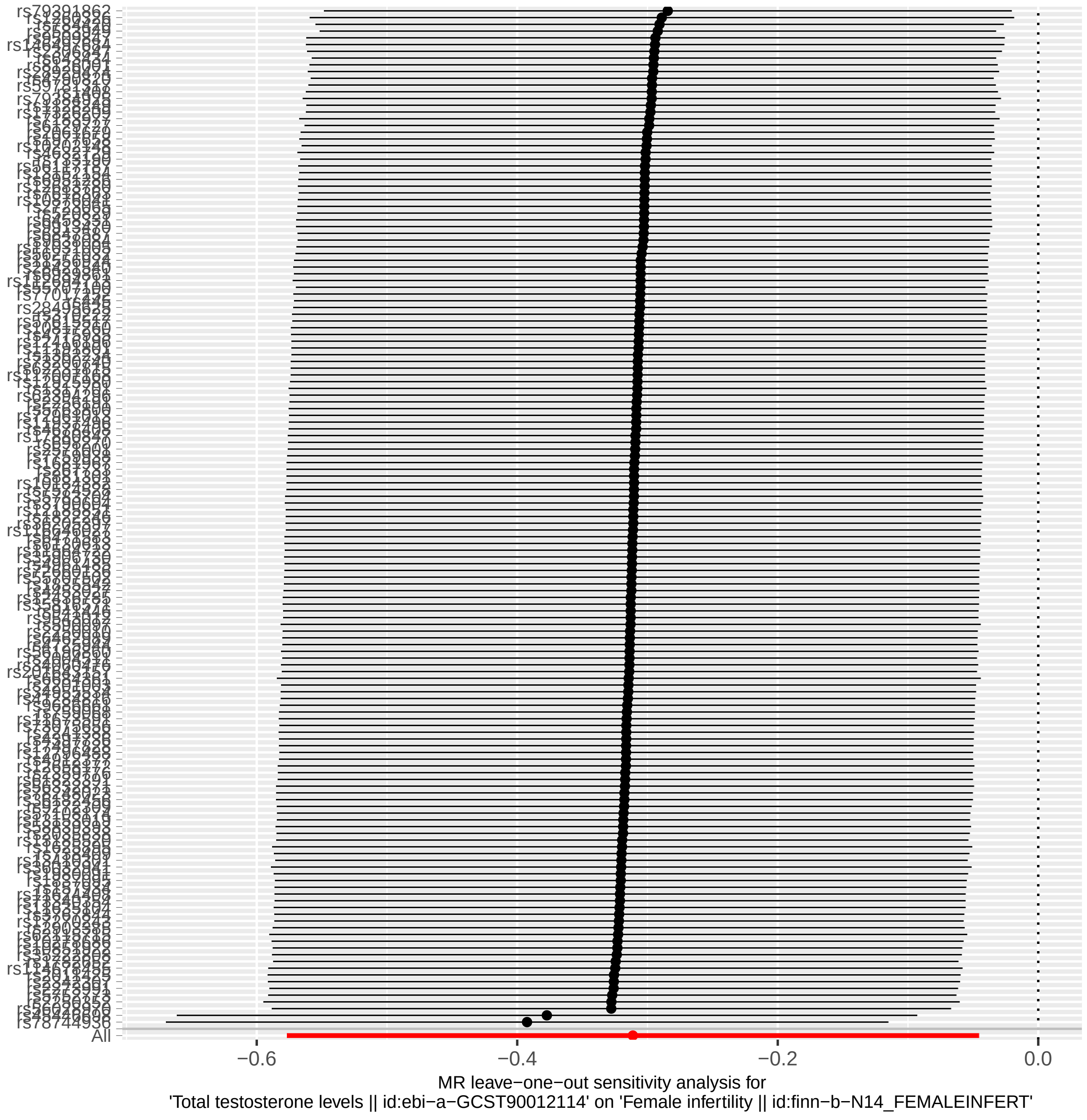
rs79391862
rs78744936
All
−0.6
−0.4
−0.2
0.0
MR leave−one−out sensitivity analysis for
'Total testosterone levels || id:ebi−a−GCST90012114' on 'Female infertility || id:finn−b−N14_FEMALEINFERT'

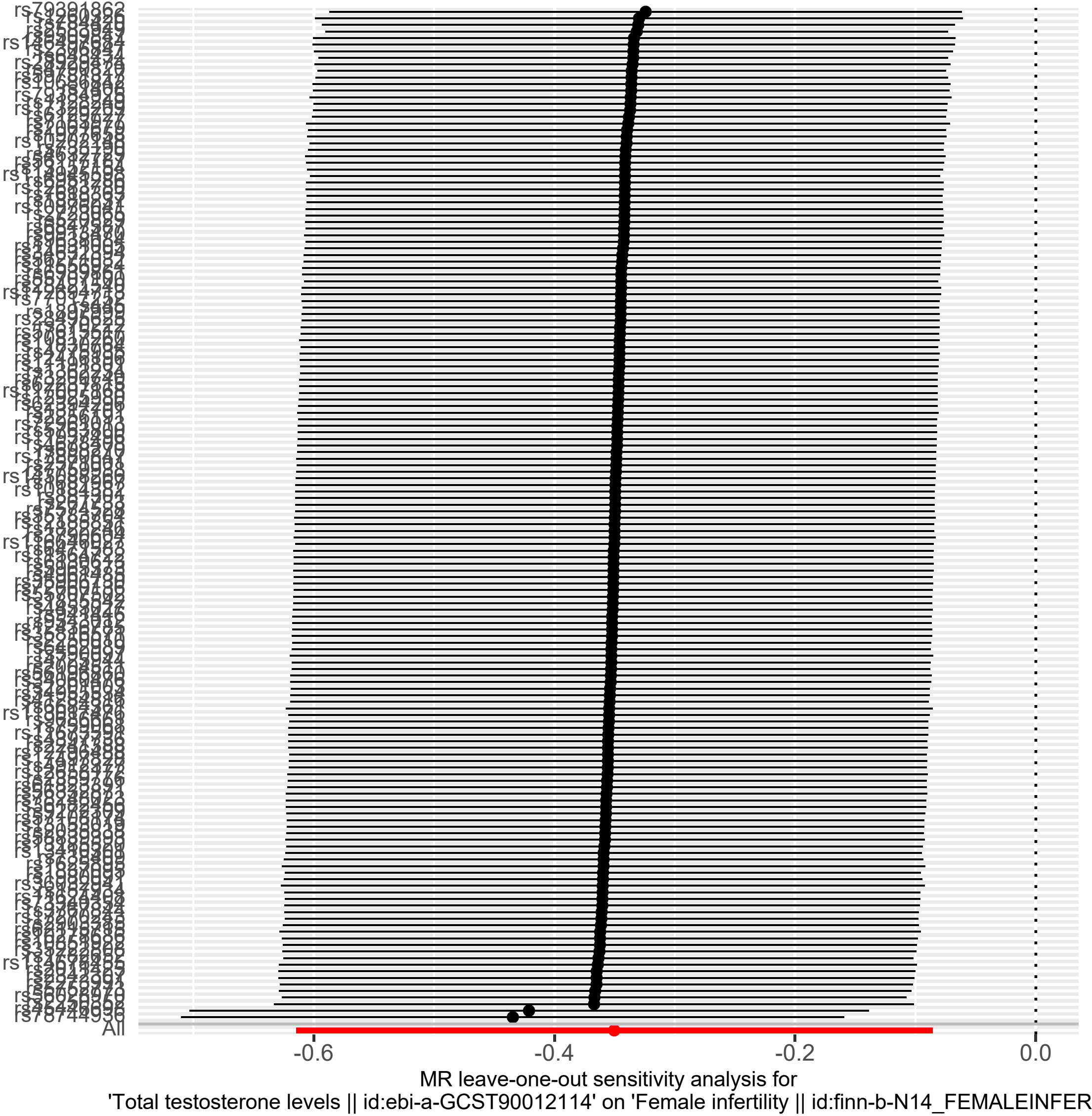
rs79201862
rs78744936
All
-0.6
-0.4
-0.2
0.0
MR leave-one-out sensitivity analysis for
'Total testosterone levels || id:ebi-a-GCST90012114' on 'Female infertility || id:finn-b-N14_FEMALEINFER

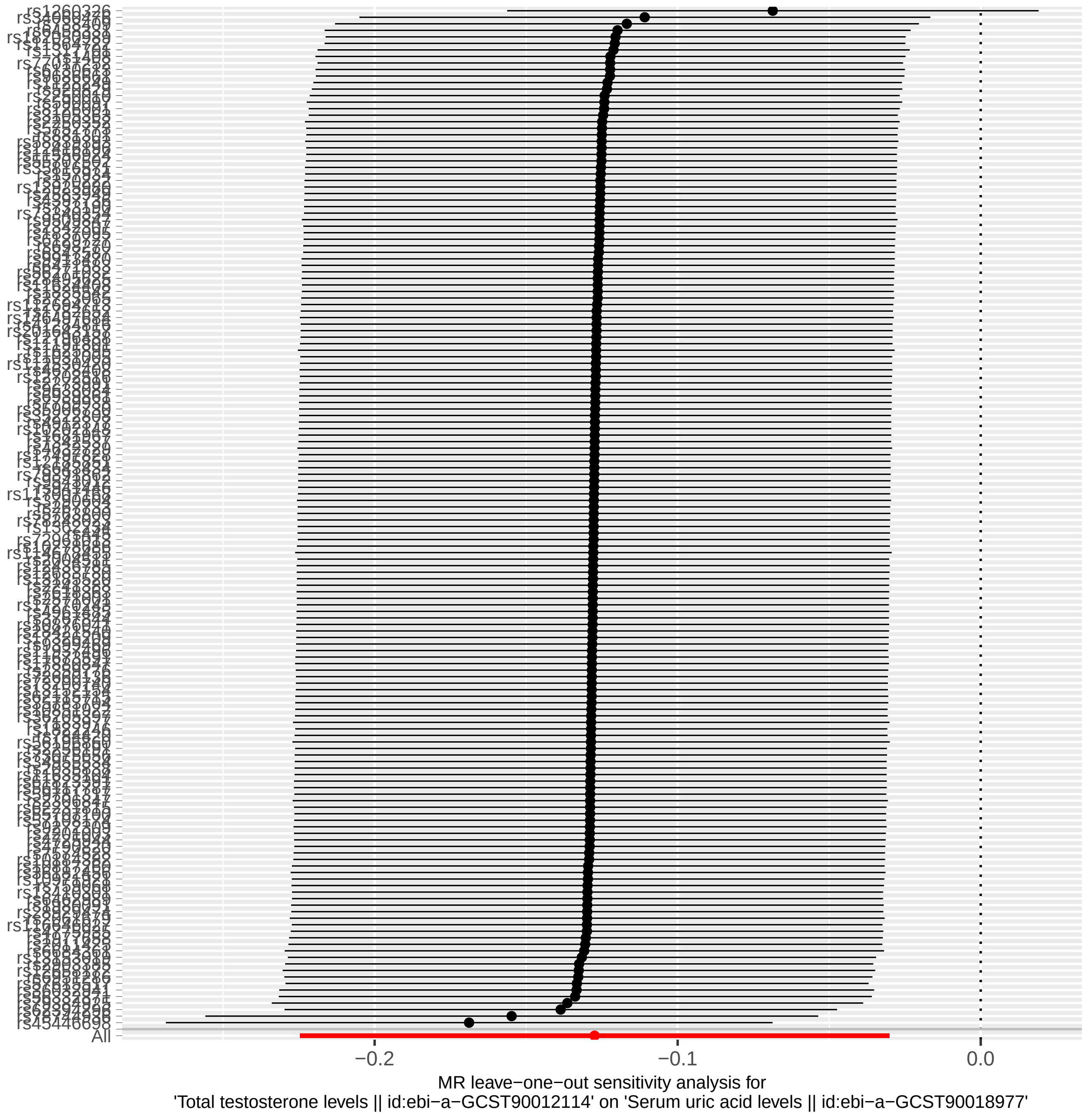
rs1260326
rs4544098
All
−0.2
−0.1
0.0
MR leave−one−out sensitivity analysis for
'Total testosterone levels || id:ebi−a−GCST90012114' on 'Serum uric acid levels || id:ebi−a−GCST90018977'

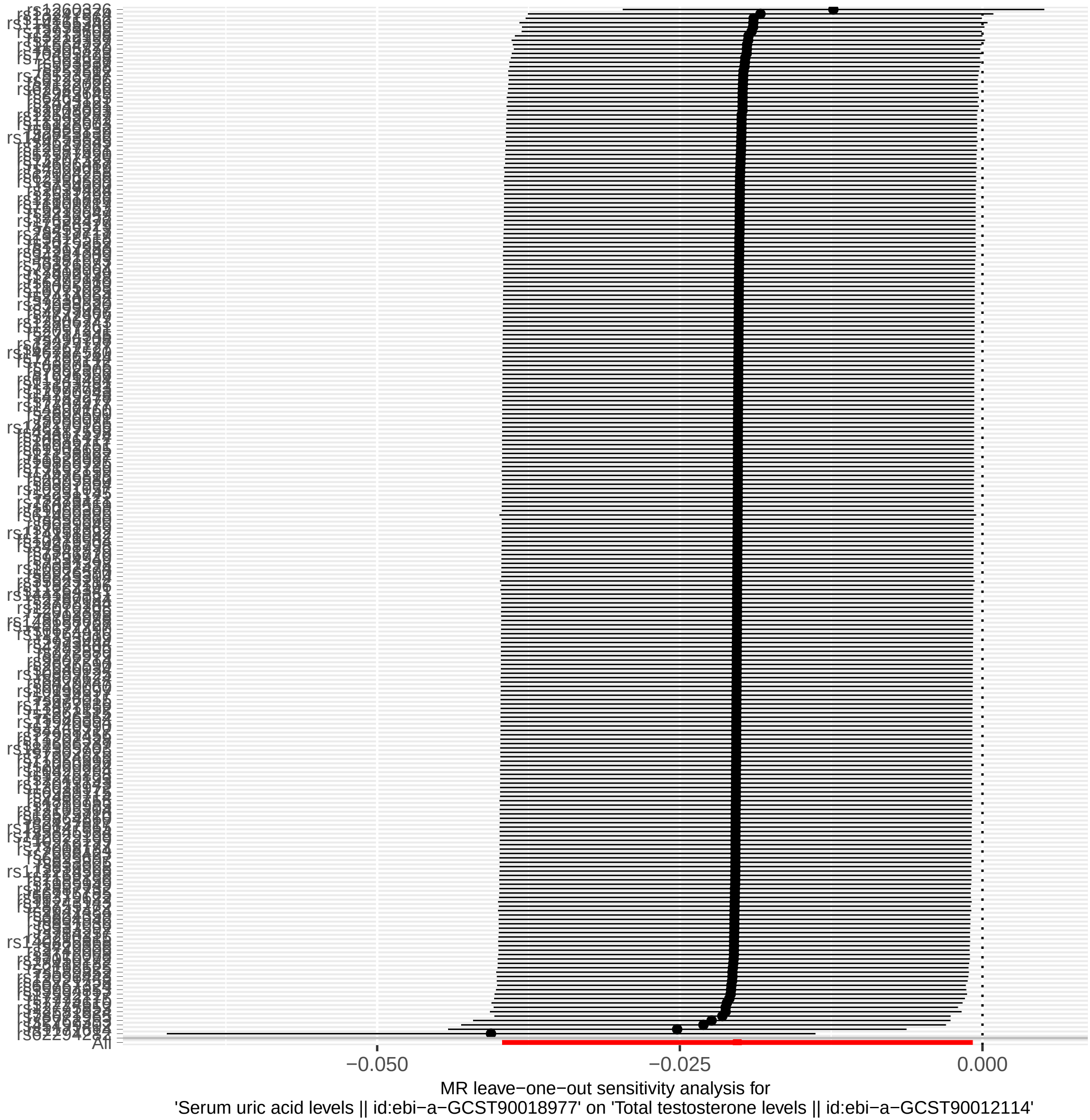
rs1260326
All
−0.050
−0.025
0.000
MR leave−one−out sensitivity analysis for
'Serum uric acid levels || id:ebi−a−GCST90018977' on 'Total testosterone levels || id:ebi−a−GCST90012114'

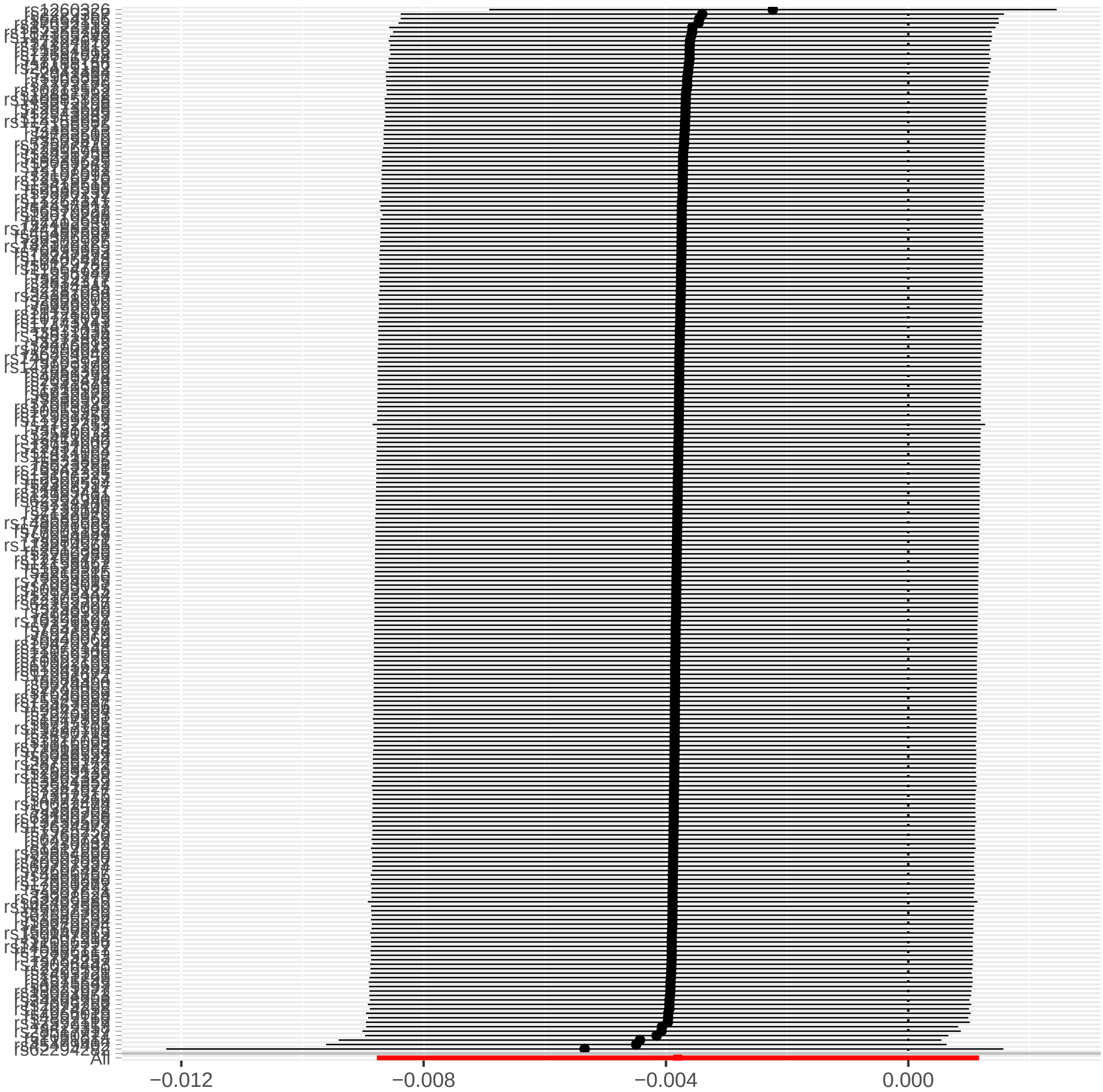

MR leave−one−out sensitivity analysis for
'Serum uric acid levels || id:ebi−a−GCST90018977' on 'Estradiol levels || id:ebi−a−GCST90012105'

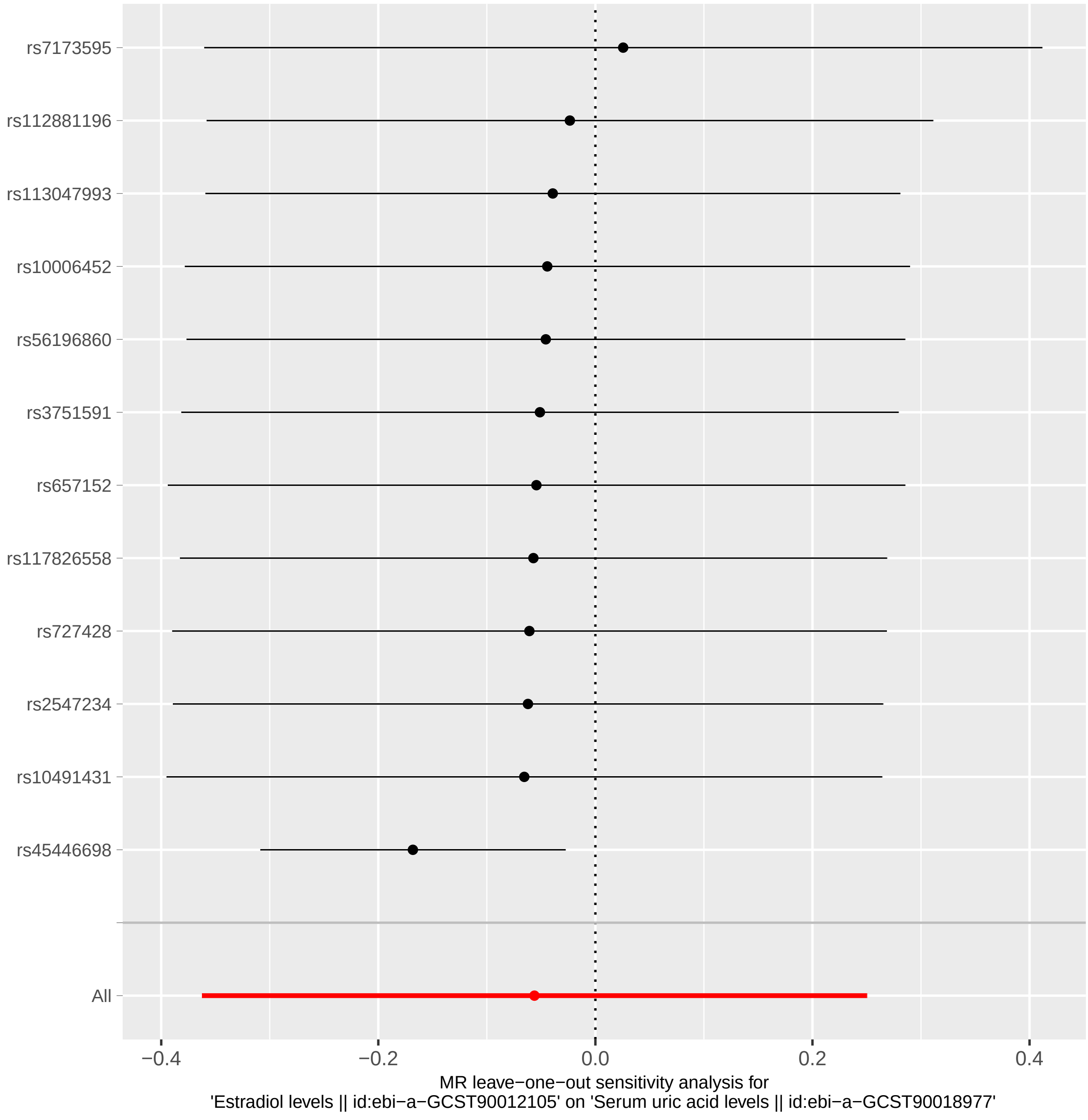
rs7173595
rs112881196
rs113047993
rs10006452
rs56196860
rs3751591
rs657152
rs117826558
rs727428
rs2547234
rs10491431
rs45446698
All
−0.4
−0.2
0.0
0.2
0.4
MR leave−one−out sensitivity analysis for
'Estradiol levels || id:ebi−a−GCST90012105' on 'Serum uric acid levels || id:ebi−a−GCST90018977'
