# Supplementary material for: Decoding connections in the European population: serum uric acid, sex hormone-binding globulin, total testosterone, estradiol, and female infertility – advanced bidirectional and mediative Mendelian randomization
Source: Front Endocrinol (Lausanne). 2024 Jun 28;15:1398600. doi: 10.3389/fendo.2024.1398600 (PMC11239382; doi:10.3389/fendo.2024.1398600)
Supplement: Supplementary file 4 [file DataSheet_4.pdf]

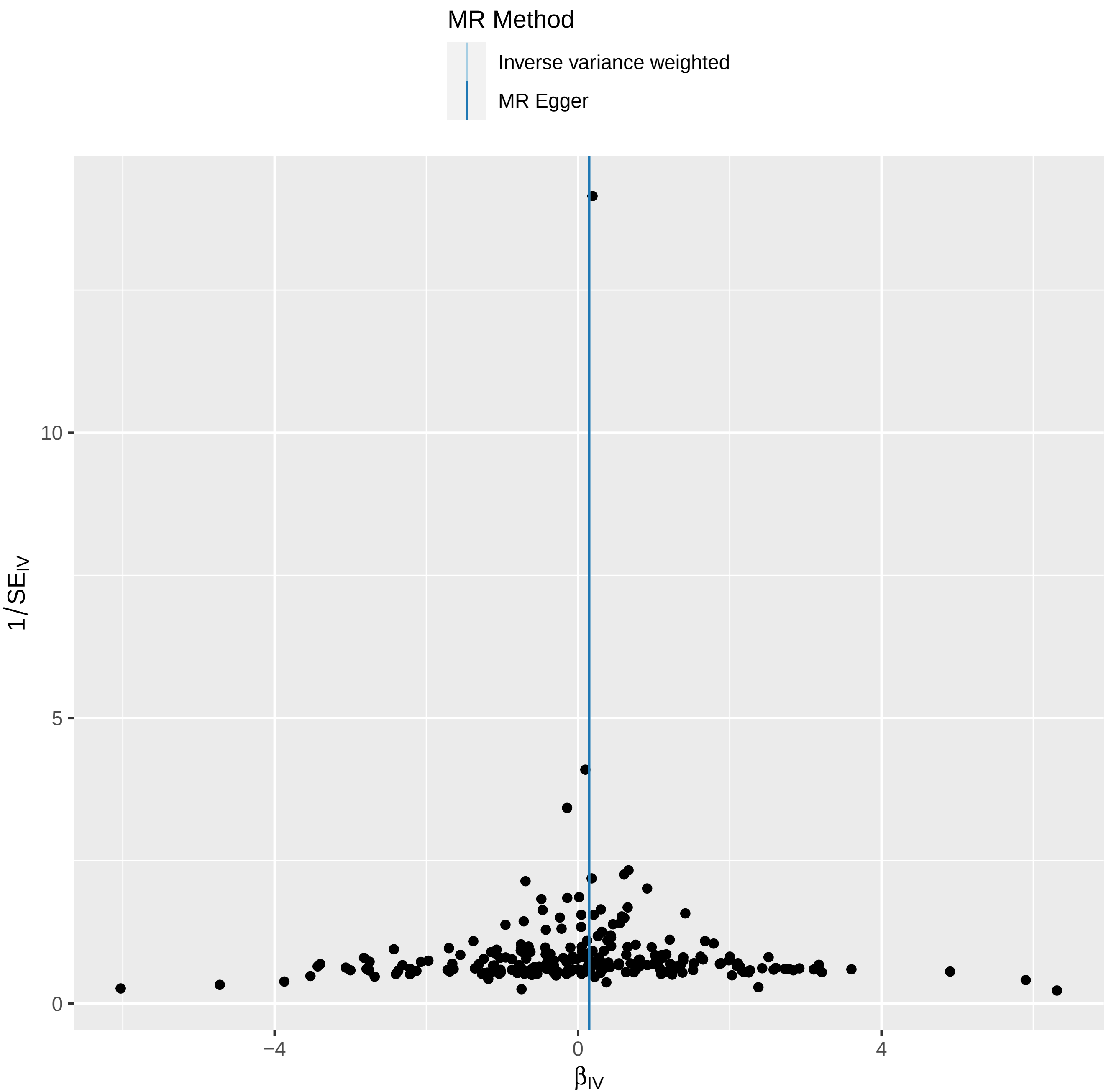
MR Method
Inverse variance weighted
MR Egger
10
5
0
1/SE_IV
−4
0
4
β_IV

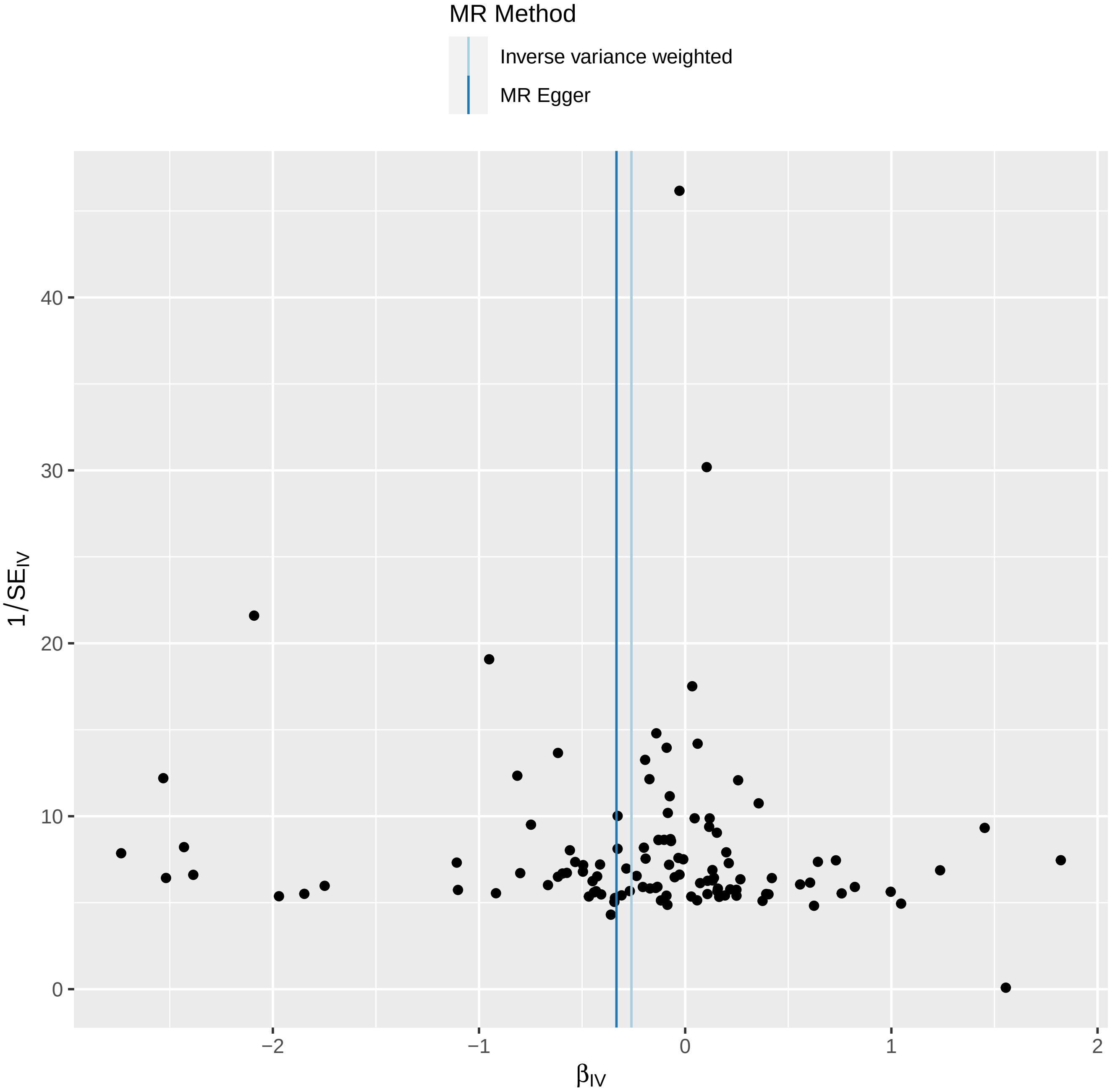

MR Method
Inverse variance weighted
MR Egger
40
30
20
10
0
1/SE_IV
−2
−1
0
1
2
β_IV

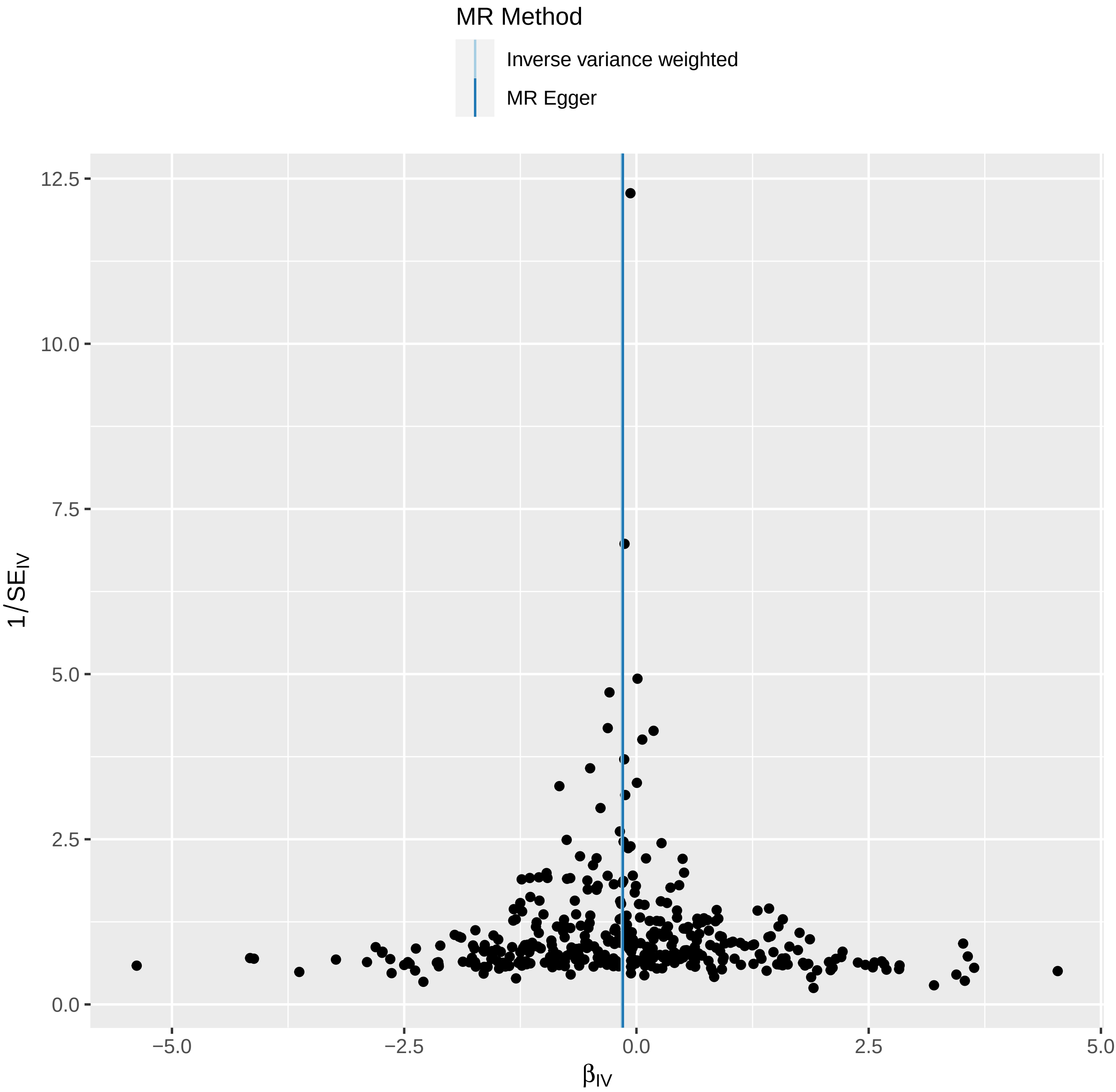
MR Method
Inverse variance weighted
MR Egger
12.5
10.0
7.5
5.0
2.5
0.0
−5.0
−2.5
0.0
2.5
5.0
1/SE_IV
β_IV

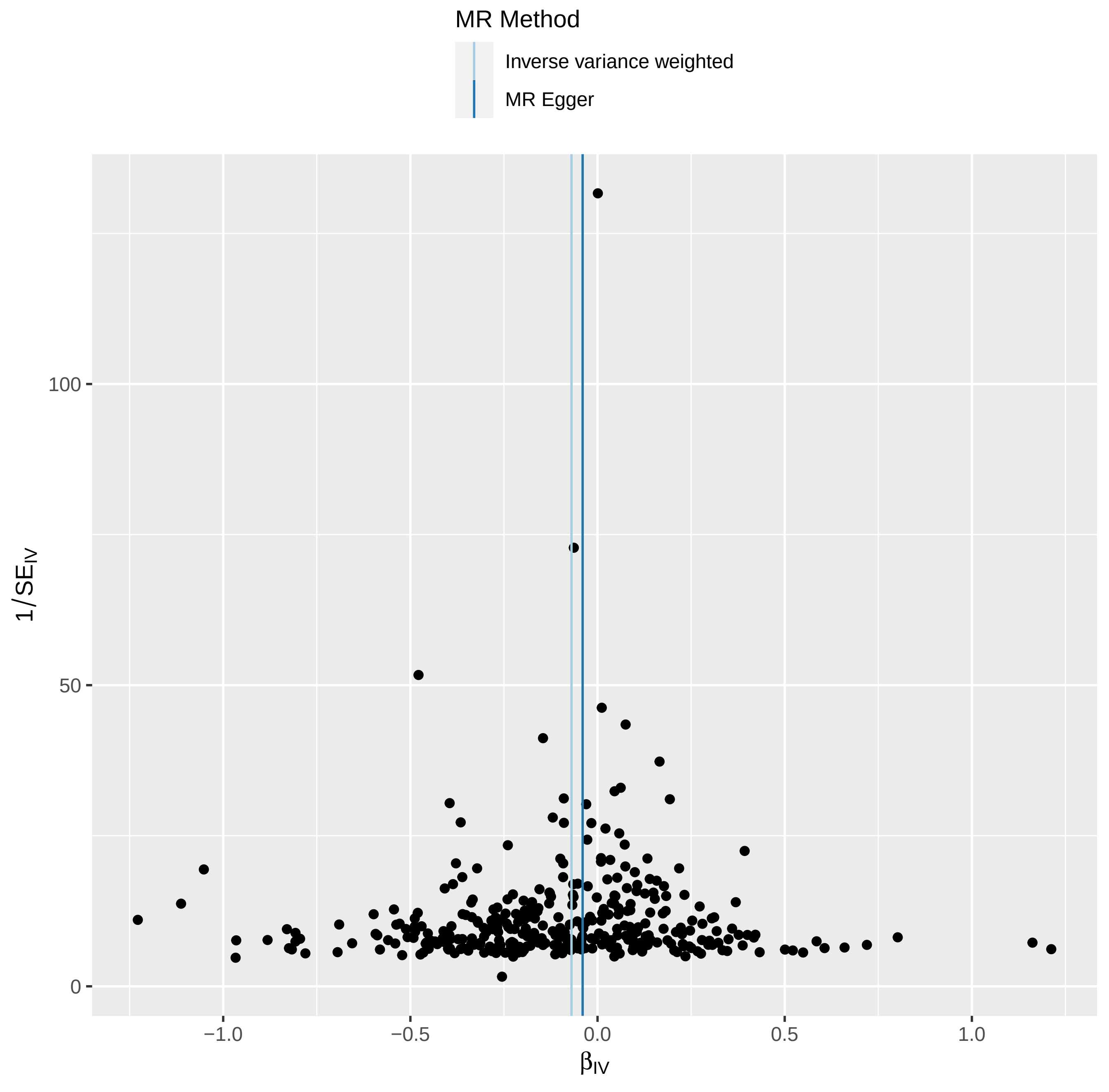

MR Method
Inverse variance weighted
MR Egger
100
50
0
−1.0
−0.5
0.0
0.5
1.0
$1/SE_{IV}$
β_{IV}

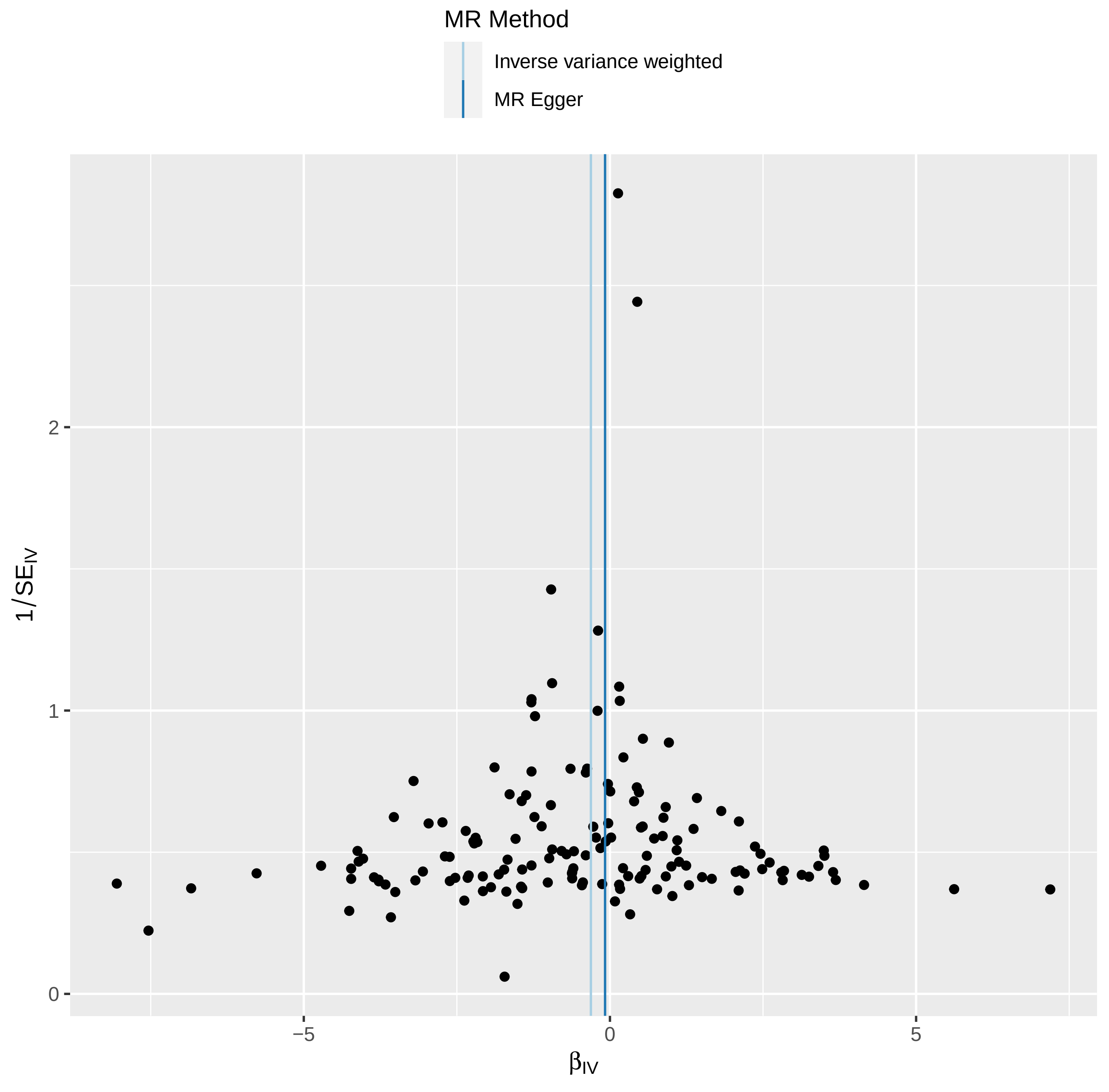
MR Method
Inverse variance weighted
MR Egger
2
1
0
1/SE_IV
−5
0
5
β_IV

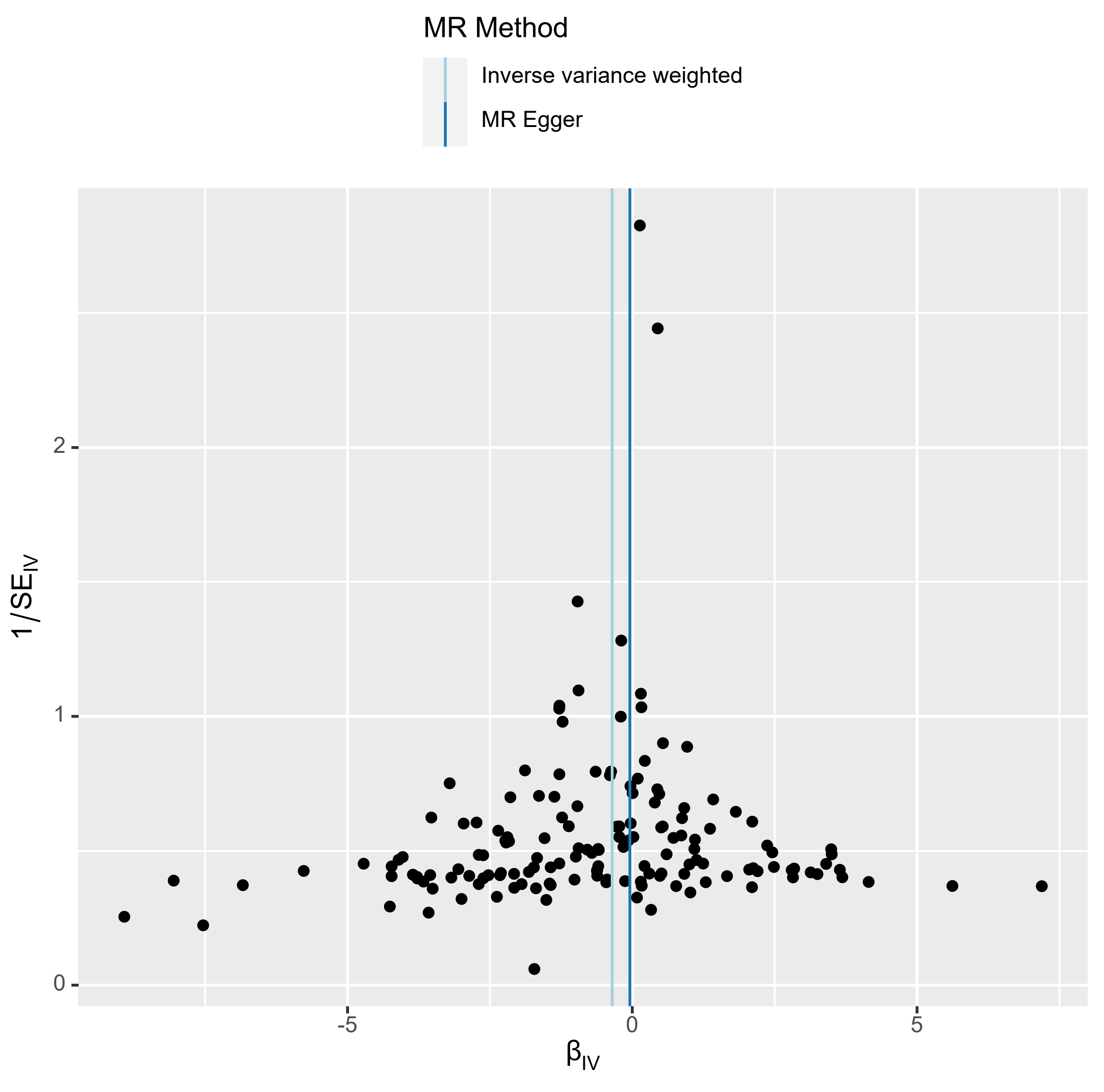

MR Method
Inverse variance weighted
MR Egger
1/SE_IV
0
1
2
-5
0
5
β_IV

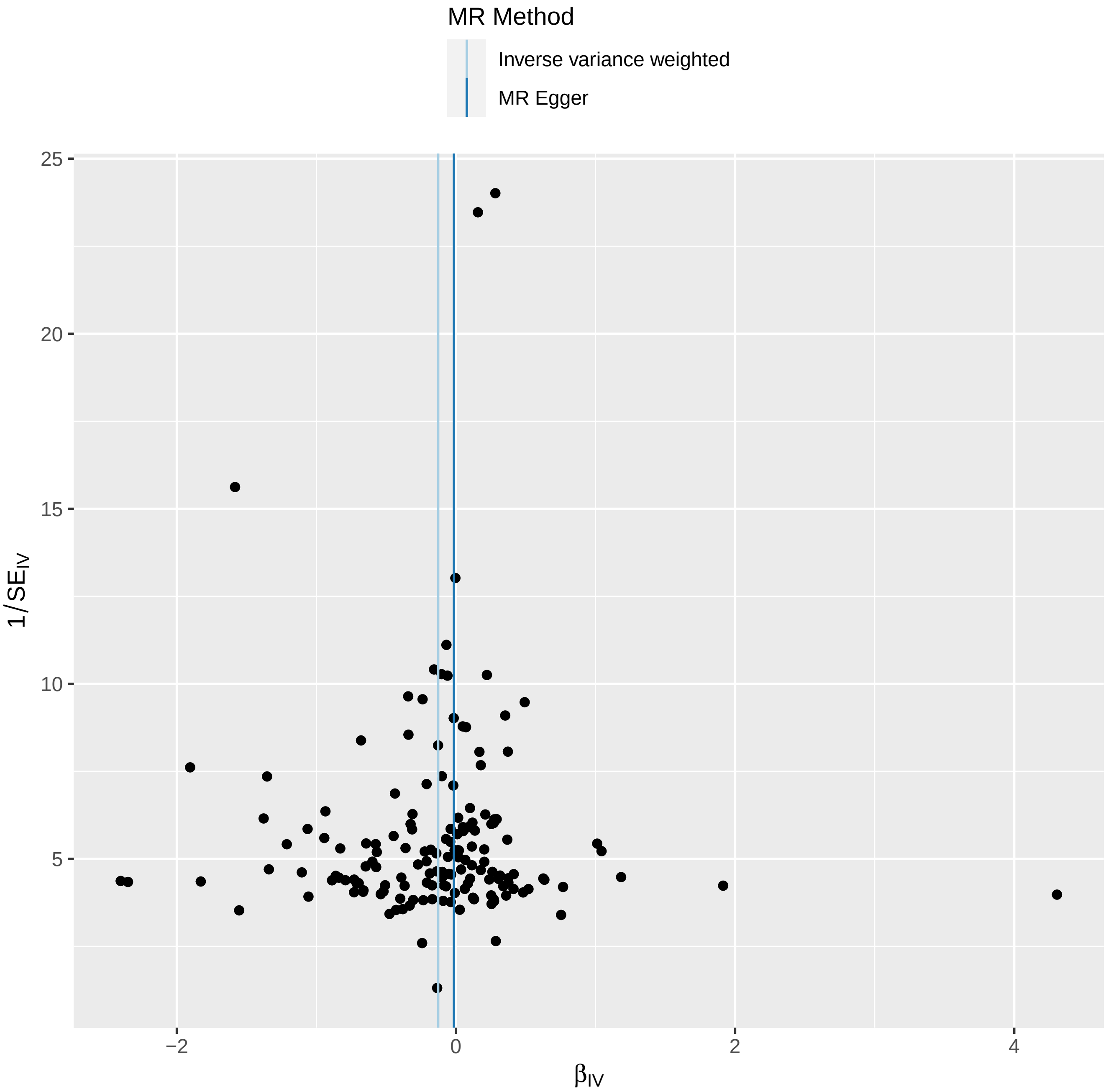
MR Method
Inverse variance weighted
MR Egger
25
20
15
10
5
1/SE_IV
−2
0
2
4
β_IV

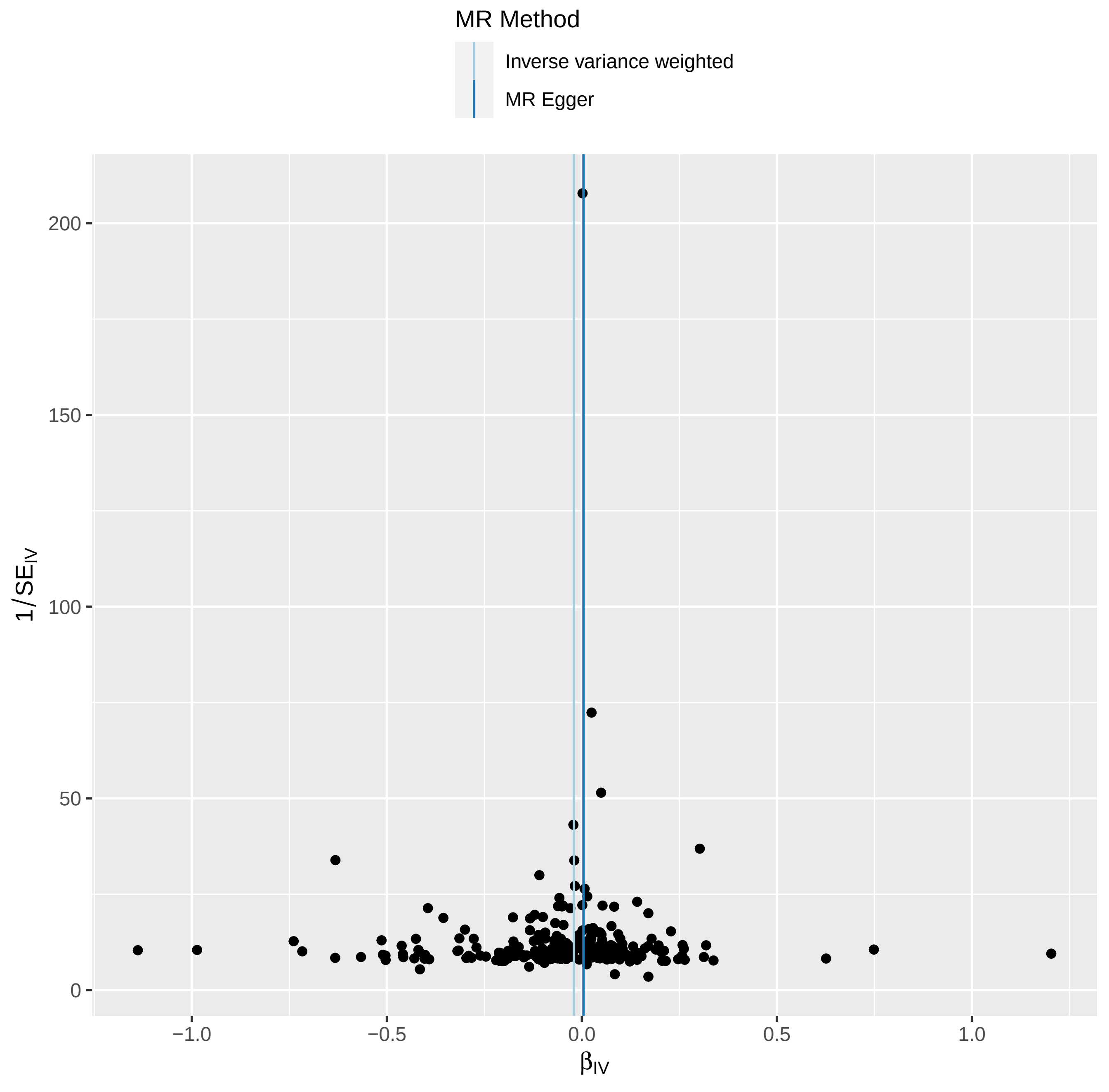
MR Method
Inverse variance weighted
MR Egger
200
150
100
50
0
1/SE_IV
−1.0
−0.5
0.0
0.5
1.0
β_IV

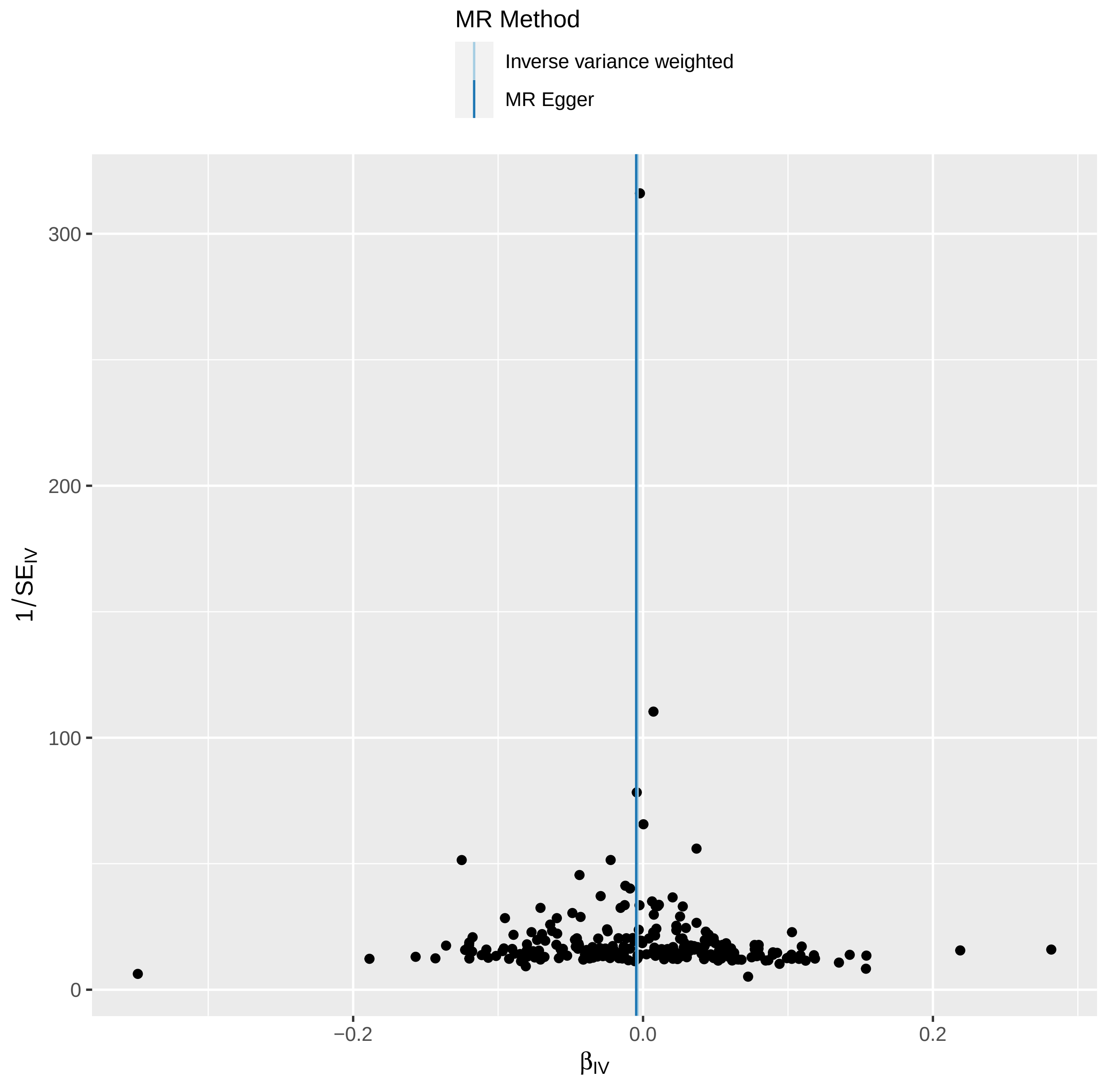

MR Method
Inverse variance weighted
MR Egger
300
200
100
0
1/SE_IV
−0.2
0.0
0.2
β_IV

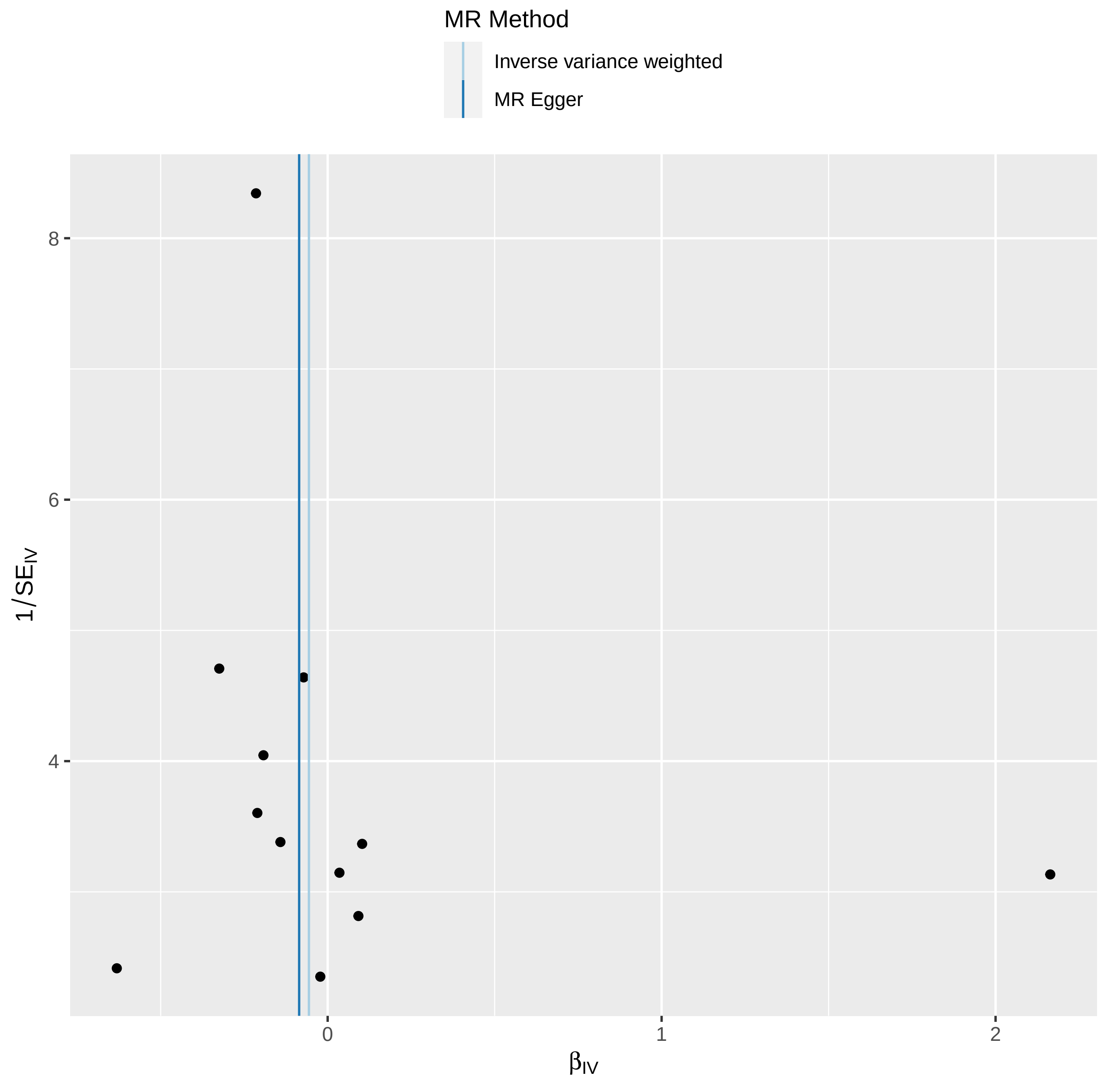

MR Method
Inverse variance weighted
MR Egger
8
6
4
1/SE$_{IV}$
0
1
2
β$_{IV}$
