# Supplementary material for: Decoding connections in the European population: serum uric acid, sex hormone-binding globulin, total testosterone, estradiol, and female infertility – advanced bidirectional and mediative Mendelian randomization
Source: Front Endocrinol (Lausanne). 2024 Jun 28;15:1398600. doi: 10.3389/fendo.2024.1398600 (PMC11239382; doi:10.3389/fendo.2024.1398600)
Supplement: Supplementary file 6 [file Table_2.doc]

**Table S11. The Association of Serum uric acid levels with Female infertility risk using five MR methods.**

| **Method** | **Beta** | **SE** | **OR** | **OR_95%CI** | **P Value** |
| --- | --- | --- | --- | --- | --- |
| **IVW** | 0.147 | 0.050 | 1.158 | 1.050-1.278 | 0.003 |
| **MR Egger** | 0.147 | 0.067 | 1.159 | 1.017-1.321 | 0.028 |
| **Weighted median** | 0.191 | 0.071 | 1.210 | 1.052-1.392 | 0.007 |
| **Simple mode** | 0.100 | 0.256 | 1.105 | 0.669-1.824 | 0.697 |
| **Weighted mode** | 0.185 | 0.067 | 1.203 | 1.055-1.372 | 0.006 |

CI, confidence interval; IVW, inverse variance-weighted; MR,

Mendelian Randomization; OR, odds ratio.

**Table S12. The Association of Serum uric acid levels with Sex hormone-binding globulin levels risk using five MR methods.**

| **Method** | **Beta** | **SE** | **OR** | **OR_95%CI** | **P Value** |
| --- | --- | --- | --- | --- | --- |
| **IVW** | -0.261 | 0.071 | 0.771 | 0.672-0.885 | 2.177e-04 |
| **MR Egger** | -0.333 | 0.126 | 0.717 | 0.560-0.917 | 0.009 |
| **Weighted median** | -0.028 | 0.021 | 0.972 | 0.933-1.013 | 0.173 |
| **Simple mode** | -0.032 | 0.047 | 0.969 | 0.884-1.062 | 0.498 |
| **Weighted mode** | -0.010 | 0.018 | 0.990 | 0.955-1.025 | 0.565 |

**Table S13. The Association of Sex hormone-binding globulin levels with Female infertility risk using five MR methods.**

| **Method** | **Beta** | **SE** | **OR** | **OR_95%CI** | **P Value** |
| --- | --- | --- | --- | --- | --- |
| **IVW** | -0.158 | 0.038 | 0.854 | 0.793-0.919 | 2.853e-05 |
| **MR Egger** | -0.146 | 0.057 | 0.864 | 0.773-0.967 | 0.011 |
| **Weighted median** | -0.075 | 0.067 | 0.928 | 0.813-1.059 | 0.268 |
| **Simple mode** | -0.088 | 0.145 | 0.916 | 0.689-1.218 | 0.547 |
| **Weighted mode** | -0.111 | 0.056 | 0.895 | 0.803-0.998 | 0.047 |

**Table S14. The Association of Sex hormone-binding globulin levels with Serum uric acid levels risk using five MR methods.**

| **Method** | **Beta** | **SE** | **OR** | **OR_95%CI** | **P Value** |
| --- | --- | --- | --- | --- | --- |
| **IVW** | -0.070 | 0.012 | 0.933 | 0.911-0.955 | 8.740e-09 |
| **MR Egger** | -0.040 | 0.018 | 0.961 | 0.927-0.996 | 0.029 |
| **Weighted median** | -0.001 | 0.007 | 0.999 | 0.985-1.014 | 0.931 |
| **Simple mode** | 0.001 | 0.050 | 1.001 | 0.908-1.103 | 0.991 |
| **Weighted mode** | -0.001 | 0.007 | 0.995 | 0.982-1.008 | 0.454 |

**Table S15. The Association of Total testosterone levels with Female infertility risk using five MR methods.**

| **Method** | **Beta** | **SE** | **OR** | **OR_95%CI** | **P Value** |
| --- | --- | --- | --- | --- | --- |
| **IVW** | -0.311 | 0.135 | 0.733 | 0.562-0.955 | 0.022 |
| **MR Egger** | -0.080 | 0.249 | 0.923 | 0.567-1.503 | 0.749 |
| **Weighted median** | 0.091 | 0.238 | 1.095 | 0.687-1.745 | 0.702 |
| **Simple mode** | -1.536e-04 | 0.463 | 0.100 | 0.403-2.478 | 9.997e-01 |
| **Weighted mode** | 0.112 | 0.245 | 1.119 | 0.692-1.808 | 0.647 |

**Table S16. The Association of Total testosterone levels with Serum uric acid levels risk using five MR methods.**

| **Method** | **Beta** | **SE** | **OR** | **OR_95%CI** | **P Value** |
| --- | --- | --- | --- | --- | --- |
| **IVW** | -0.127 | 0.050 | 0.880 | 0.799-0.970 | 0.010 |
| **MR Egger** | -0.014 | 0.095 | 0.986 | 0.819-1.187 | 0.880 |
| **Weighted median** | 0.017 | 0.028 | 1.017 | 0.961-1.074 | 0.556 |
| **Simple mode** | 0.009 | 0.066 | 1.009 | 0.886-1.149 | 0.894 |
| **Weighted mode** | 0.113 | 0.037 | 1.119 | 1.040-1.205 | 0.003 |

**Table S17. The Association of Serum uric acid levels with Total testosterone levels risk using five MR methods.**

| **Method** | **Beta** | **SE** | **OR** | **OR_95%CI** | **P Value** |
| --- | --- | --- | --- | --- | --- |
| **IVW** | -0.020 | 0.010 | 0.980 | 0.961-0.999 | 0.041 |
| **MR Egger** | 0.004 | 0.013 | 1.004 | 0.979-1.030 | 0.759 |
| **Weighted median** | 0.002 | 0.005 | 1.001 | 0.993-1.011 | 0.716 |
| **Simple mode** | -0.036 | 0.021 | 0.964 | 0.925-1.006 | 0.091 |
| **Weighted mode** | 0.005 | 0.005 | 1.005 | 0.995-1.014 | 0.353 |

**Table S18. The Association of Serum uric acid levels with Estradiol levels risk using five MR methods.**

| **Method** | **Beta** | **SE** | **OR** | **OR_95%CI** | **P Value** |
| --- | --- | --- | --- | --- | --- |
| **IVW** | -0.004 | 0.003 | 0.996 | 0.991-1.001 | 0.133 |
| **MR Egger** | -0.005 | 0.003 | 0.995 | 0.989-1.002 | 0.162 |
| **Weighted median** | -0.002 | 0.003 | 0.998 | 0.992-1.004 | 0.507 |
| **Simple mode** | 0.009 | 0.012 | 1.009 | 0.985-1.033 | 0.488 |
| **Weighted mode** | -3.217e-4 | 0.003 | 1.000 | 0.994-1.005 | 0.910 |

**Table S19. The Association of Estradiol levels with Serum uric acid levels risk using five MR methods.**

| **Method** | **Beta** | **SE** | **OR** | **OR_95%CI** | **P Value** |
| --- | --- | --- | --- | --- | --- |
| **IVW** | -0.056 | 0.156 | 0.945 | 0.696-1.284 | 0.719 |
| **MR Egger** | -0.085 | 0.404 | 0.918 | 0.416-2.026 | 0.837 |
| **Weighted median** | -0.211 | 0.092 | 0.810 | 0.676-0.970 | 0.022 |
| **Simple mode** | -0.150 | 0.121 | 0.861 | 0.679-1.091 | 0.242 |
| **Weighted mode** | -0.203 | 0.104 | 0.816 | 0.665-1.001 | 0.078 |

**Table S20 Cochran’s Q tests for heterogeneity from MR-IVW analyses.**

| **Exposure** | **Outcome** | **Q** | **df** | **P-value** |
| --- | --- | --- | --- | --- |
| **Serum uric acid levels** | **Female infertility** | **229.664** | **227** | **0.438** |
| **Serum uric acid levels** | **Sex hormone-binding globulin levels** | **5648.932** | **110** | **0** |
| **Serum uric acid levels** | **Total testosterone levels** | **2052.540** | **233** | **4.480e-288** |
| **Serum uric acid levels** | **Estradiol levels** | **310.243** | **233** | **5.278e-04** |
| **Female infertility** | **Serum uric acid levels** | **NA** | **NA** | **NA** |
| **Sex hormone-binding globulin levels** | **Serum uric acid levels** | **4181.308** | **356** | **0** |
| **Total testosterone levels** | **Serum uric acid levels** | **2396.029** | **155** | **0** |
| **Estradiol levels** | **Serum uric acid levels** | **54.911** | **11** | **8.047e-08** |
| **Sex hormone-binding globulin levels** | **Female infertility** | **380.858** | **358** | **0.194** |
| **Total testosterone levels** | **Female infertility** | **169.638** | **150** | **0.130** |

**Table S21 MR-Egger intercept tests for horizontal pleiotropy.**

| **Exposure** | **Outcome** | **Egger intercept** | **SE** | **P-value** |
| --- | --- | --- | --- | --- |
| **Serum uric acid levels** | **Female infertility** | **-2.374e-05** | **0.002** | **0.991** |
| **Serum uric acid levels** | **Sex hormone-binding globulin levels** | **0.002** | **0.003** | **0.489** |
| **Serum uric acid levels** | **Total testosterone levels** | **-0.001** | **3.988e-04** | **0.005** |
| **Serum uric acid levels** | **Estradiol levels** | **4.351e-05** | **1.037e-04** | **0.676** |
| **Female infertility** | **Serum uric acid levels** | **NA** | **NA** | **NA** |
| **Sex hormone-binding globulin levels** | **Serum uric acid levels** | **-0.001** | **0.001** | **0.030** |
| **Total testosterone levels** | **Serum uric acid levels** | **-0.002** | **0.001** | **0.163** |
| **Estradiol levels** | **Serum uric acid levels** | **3.302e-04** | **0.004** | **0.938** |
| **Sex hormone-binding globulin levels** | **Female infertility** | **-5.368e-04** | **0.002** | **0.782** |
| **Total testosterone levels** | **Female infertility** | **-4.003e-03** | **0.004** | **0.269** |
